# Medication Optimization Protocol Efficacy for Geriatric Inpatients: A Randomized Clinical Trial

**DOI:** 10.1001/jamanetworkopen.2024.23544

**Published:** 2024-07-30

**Authors:** Kenya Ie, Masanori Hirose, Tsubasa Sakai, Iori Motohashi, Mari Aihara, Takuya Otsuki, Ayako Tsuboya, Hiroshi Matsumoto, Hikari Hashi, Eisuke Inoue, Masaki Takahashi, Eiko Komiya, Yuka Itoh, Reiko Machino, Tomoya Tsuchida, Steven M. Albert, Yoshiyuki Ohira, Chiaki Okuse

**Affiliations:** 1 Department of General Internal Medicine, St Marianna University School of Medicine, Kanagawa, Japan; 2 Department of General Internal Medicine, Kawasaki Municipal Tama Hospital, Kanagawa, Japan; 3 Department of Pharmacy, Kawasaki Municipal Tama Hospital, Kanagawa, Japan; 4 Showa University Research Administration Center, Showa University, Tokyo, Japan; 5 Division of Medical Informatics, St Marianna University School of Medicine, Kanagawa, Japan; 6 Department of Behavioral and Community Health Sciences, University of Pittsburgh Graduate School of Public Health, Pittsburgh, Pennsylvania

## Abstract

**Importance:**

There is currently no consensus on clinically effective interventions for polypharmacy among older inpatients.

**Objective:**

To evaluate the effect of multidisciplinary team-based medication optimization on survival, unscheduled hospital visits, and rehospitalization in older inpatients with polypharmacy.

**Design, Setting, and Participants:**

This open-label randomized clinical trial was conducted at 8 internal medicine inpatient wards within a community hospital in Japan. Participants included medical inpatients 65 years or older who were receiving 5 or more regular medications. Enrollment took place between May 21, 2019, and March 14, 2022. Statistical analysis was performed from September 2023 to May 2024.

**Intervention:**

The participants were randomly assigned to receive either an intervention for medication optimization or usual care including medication reconciliation. The intervention consisted of a medication review using the STOPP (Screening Tool of Older Persons’ Prescriptions)/START (Screening Tool to Alert to Right Treatment) criteria, followed by a medication optimization proposal for participants and their attending physicians developed by a multidisciplinary team. On discharge, the medication optimization summary was sent to patients’ primary care physicians and community pharmacists.

**Main Outcomes and Measures:**

The primary outcome was a composite of death, unscheduled hospital visits, and rehospitalization within 12 months. Secondary outcomes included the number of prescribed medications, falls, and adverse events.

**Results:**

Between May 21, 2019, and March 14, 2022, 442 participants (mean [SD] age, 81.8 [7.1] years; 223 [50.5%] women) were randomly assigned to the intervention (n = 215) and usual care (n = 227). The intervention group had a significantly lower percentage of patients with 1 or more potentially inappropriate medications than the usual care group at discharge (26.2% vs 33.0%; adjusted odds ratio [OR], 0.56 [95% CI, 0.33-0.94]; *P* = .03), at 6 months (27.7% vs 37.5%; adjusted OR, 0.50 [95% CI, 0.29-0.86]; *P* = .01), and at 12 months (26.7% vs 37.4%; adjusted OR, 0.45 [95% CI, 0.25-0.80]; *P* = .007). The primary composite outcome occurred in 106 participants (49.3%) in the intervention group and 117 (51.5%) in the usual care group (stratified hazard ratio, 0.98 [95% CI, 0.75-1.27]). Adverse events were similar between each group (123 [57.2%] in the intervention group and 135 [59.5%] in the usual care group).

**Conclusions and Relevance:**

In this randomized clinical trial of older inpatients with polypharmacy, the multidisciplinary deprescribing intervention did not reduce death, unscheduled hospital visits, or rehospitalization within 12 months. The intervention was effective in reducing the number of medications with no significant adverse effects on clinical outcomes, even among older inpatients with polypharmacy.

**Trial Registration:**

UMIN Clinical Trials Registry: UMIN000035265

## Key Points

**Question:**

What is the effect of multidisciplinary team–based medication optimization on clinical end points in older inpatients with polypharmacy?

**Findings:**

In this randomized clinical trial that included 442 patients, the intervention did not reduce death, unscheduled hospital visits, or rehospitalization. The intervention was safe and effective in reducing the number of medications and potentially inappropriate medications.

**Meaning:**

These results corroborate the safety of deprescribing interventions in older inpatients with polypharmacy, consistent with previous studies; however, the inability of this health care–centered intervention to affect health outcomes may suggest the need for patient-centered interventions.

## Teaser

This randomized clinical trial investigates whether multidisciplinary team-based medication optimization has an effect on survival, unscheduled hospital visits, and rehospitalization among older inpatients with polypharmacy.

## Introduction

A substantial portion of older adults are subject to polypharmacy, defined as the concurrent use of multiple medications, a trend that has been on the rise.^1,2^ Use of multiple medications is associated with a high risk of physical and cognitive disability,^3^ emergency department visits and hospitalizations,^4^ and mortality.^5^ Furthermore, polypharmacy is associated with nearly doubling overall health care costs and tripling pharmacy expenses, as indicated by the 2017 Medical Expenditure Panel Survey data.^6^

Various interventions have been developed to mitigate the negative impact of polypharmacy in the last few decades. Interventions with a focus on reducing specific medications^7,8^ or potentially inappropriate medications (PIMs) (listed in explicit criteria such as the Beers criteria,^9^ which identifies PIMs in older adults, or STOPP [Screening Tool of Older Persons’ Prescriptions]/START [Screening Tool to Alert to Right Treatment] criteria,^10^ which outline medications to stop and start in older patients), have been a mainstay of such efforts. More recently, patient-centered deprescribing has become the focus of interventions and research in the field.^11^ It involves an implicit criteria-based approach to identify and discontinue drugs for which harms outweigh benefits. However, recent systematic reviews regarding the effect of interventions to reduce polypharmacy, including both the explicit and implicit criteria-based approaches, are not consistent in demonstrating their benefits on clinically important end points.^12,13,14,15^

Several factors may explain the inconsistent results observed in previous systematic reviews, including insufficient statistical power and heterogeneity in meta-analyses. Furthermore, the effect of interventions using explicit criteria could be potentially compromised by adverse drug events involving medications not listed in these criteria.^16^ A Cochrane review also highlighted methodological limitations of previous clinical trials, such as short follow-up, which might obscure the true effect of deprescribing protocols.^13^ Addressing these gaps, our intervention uses both explicit and implicit criteria to promote prescribing optimization, which we assessed over 12 months. This study aims to evaluate the efficacy of multidisciplinary team-based medication optimization on survival, unscheduled hospital visits, and rehospitalization in older inpatients with polypharmacy.

## Methods

### Study Design

We conducted a single-center, open-label, randomized clinical trial with a 2-group parallel design. The study was approved by the St Marianna University School of Medicine institutional ethical committee. This manuscript was written in accordance with the Consolidated Standards of Reporting Trials (CONSORT) reporting guideline^17^ and the Template for Intervention Description and Replication (TIDieR) checklist.^18^ Further details of the methods are available in the trial protocol (Supplement 1).^19^

### Participants

Medical inpatients admitted to a community hospital were eligible to participate if they were 65 years or older, receiving 5 or more regular medications, deemed eligible to take oral medication by their attending physician, and whose duration of hospitalization was expected to be 1 week or longer. Patients were excluded if the attending physician disagreed on their participation, or if they had a life expectancy of less than 1 month based on their attending physician’s clinical judgement. A regular medication was defined as any orally administered prescription medication that was documented in the participant’s medical record with a duration of 28 days or longer at the time of admission. Medications categorized as *as needed* were excluded from the medication counts (eMethods in Supplement 2). We obtained written informed consent from all participants or their next of kin.

### Randomization and Masking

We stratified eligible participants by age group (65-74 years, 75-84 years, and ≥85 years) and randomly allocated them within strata to the intervention and usual care groups by block randomization with block sizes of 4. The block size was concealed until the completion of the trial and was known only to the statistical analysts who were not involved in enrollment and data acquisition. Physicians, pharmacists, patients, and/or their next of kin were not masked to the allocation. All primary outcome data and part of secondary outcomes including quality-of-life score, level of long-term care required, adverse events, and falls were collected through the bimonthly telephone interview assessments by designated research assistants who were masked to group allocation. Prescription drug data were extracted either from the participant’s medical record handbook or electronic health record by research assistants using a predefined procedure.

### Trial Procedures

Given the medical risk of older patients, who are at a higher risk of medication-related adverse events, we deemed it ethically unacceptable to withhold medication reconciliation. Therefore, usual care included medication reconciliation conducted by ward-based pharmacists at admission. For participants in the intervention group, a multidisciplinary deprescribing team, composed of a physician and a pharmacist, promptly initiated the intervention within 48 hours of allocation. All members of the intervention team underwent standardized guidance and training in advance. The structure of the intervention included: (1) baseline data collection, including age, sex, previous medical history, comorbid conditions, smoking status, physical measurements on admission (height, weight and vital signs), estimated glomerular filtration rate (eGFR), serum sodium and potassium level, and regularly prescribed medications, conducted through medical record review; (2) preliminary medication optimization proposal using a clinical-decision support system; baseline data were entered into a computer-based clinical-decision support system developed specifically for this trial by the deprescribing team using a Microsoft Excel spreadsheet. Using these data, the clinical-decision support system generated a preliminary list of potentially inappropriate prescriptions as well as prescribing omissions in line with STOPP/START criteria (version 2)^20^; and (3) medication optimization protocol-based team discussion; the deprescribing team reviewed the draft proposal and embarked on a step-by-step discussion, adhering to the medication optimization protocol; this discussion followed a specific algorithm, as proposed by Scott et al^11,19^: (1) assessment of medication indication; (2) balancing benefits and harms; (3) evaluation of symptomatic medications; and (4) evaluation of preventive medications.

Following these steps, the medication optimization plan was explained and discussed with the participant or their next of kin. With the participant’s consent, the team recommended the medication optimization plan, including its rationale, to the attending physician. The decision to accept or decline the proposal was at the discretion of their attending physician. A summary of the medication optimization, including reasons for modifications and relevant precautions, was conveyed to the participant’s primary care physician and community pharmacists upon discharge, ensuring continuity of care. More details are provided in the trial protocol (Supplement 1).^19^

### Outcomes

The primary outcome was a composite of all-cause death, unscheduled hospital visits, and rehospitalization within 48 weeks of enrollment. Secondary outcomes included each of the primary outcome, number of regular medications and PIMs based on STOPP/START criteria version 2,^20^ level of long-term care required, health-related quality of life (measured using EuroQol 5 dimensions 3-levels [EQ5D-3L]),^21,22^ adverse events, falls, and all-cause death during initial hospitalization (eMethods in Supplement 2). Events were tracked in regular follow-up telephone interviews performed by trained research assistants masked to group allocation. Number of regular medications and PIMs at discharge, 24 weeks, and 48 weeks were collected using medication lists included in the participant’s medical record handbook or electronic medical record and consecutively adjudicated with medication lists sent from the relevant community pharmacist. Level of long-term care required and health-related quality of life^21,22^ were assessed at baseline, hospital discharge, 24 weeks, and 48 weeks based on the follow-up telephone interview according to the prespecified questionnaire. All adverse events, regardless of their potential relevance to the intervention, were recorded during initial hospital admission and follow-up period. Serious adverse events included all-cause death and events that resulted in persistent disability or hospital admission (eMethods in Supplement 2). The relevance to the intervention was assessed by consensus among the deprescribing team and reviewed by the institutional data and safety monitoring board.

### Statistical Analysis

With 250 participants in each group, an assumption of primary end point rates of 30% and 40% within 48 weeks in the intervention and control groups, respectively, and a true hazard ratio (HR) of 0.75,^23,24^ statistical power was 80% (2-sided type 1 error level of .05, with a 48-week drop-out rate of 15%). The efficacy end points were analyzed according to modified intention-to-treat principle. All study participants were included in the analysis of primary and secondary end points, except those found to be ineligible after enrollment. Survival functions of the primary end point were analyzed by Kaplan-Meier method and compared using a log-rank test stratified by age group. Two-sided *P* < .05 was considered statistically significant. Age group-stratified HR and 95% CI across groups was estimated using Cox proportional hazards model. We also conducted prespecified subgroup analyses of the primary end point for indicator diseases (including heart failure, pneumonia, diabetes, ischemic stroke, and urinary tract infection) and indicator drug classes (antiplatelets, antihypertensives, antidiabetics, and sedatives) for exploratory, hypothesis-generating purposes. Furthermore, a per-protocol analysis, using data from participants for whom at least 1 deprescribing was proposed and at least 1 of the proposed drugs was actually reduced at discharge, was conducted to assess the effect of the acceptance rate of the proposal. Patients who died during initial hospitalization were excluded from the per-protocol analysis, as the acceptance of the proposal could not be determined. All statistical analyses were performed using R version 4.2.2 software (R Project for Statistical Computing) from September 2023 to May 2024.

## Results

**Figure 1.**
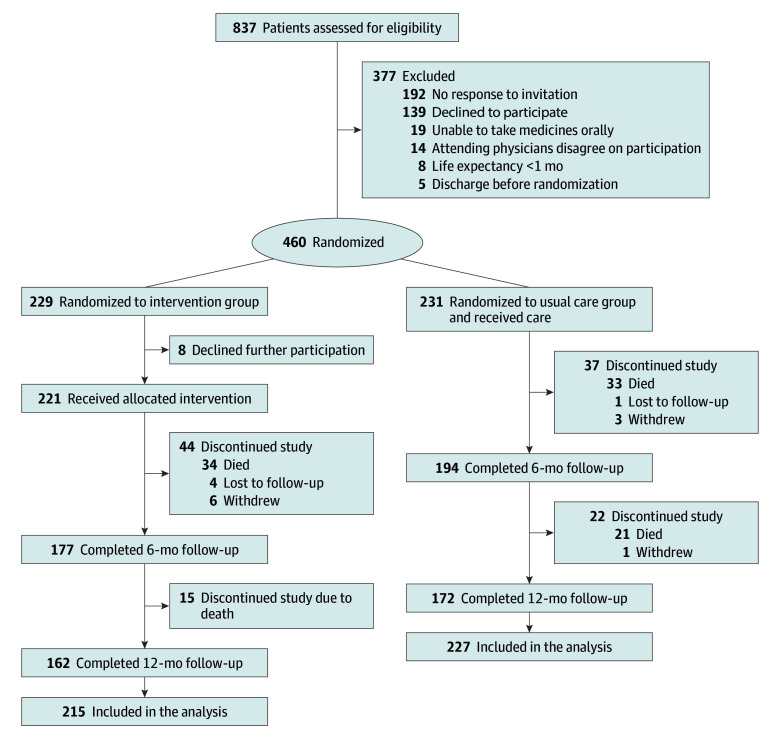
Flow of Participants Through the Study

Between May 21, 2019, and March 14, 2022, a total of 465 participants were enrolled and 460 randomized to the intervention or usual care. During follow-up, 18 patients withdrew from the study and 442 patients were included in the analysis (mean [SD] age, 81.8 [7.1] years; 223 [50.5%] women; 215 in the intervention group and 227 in the usual care group). Five patients were lost to follow-up, and 103 (23.3%) died within 12 months of follow-up (Figure 1). Baseline characteristics were similar in both groups, with the exception of a higher prevalence of smoking among participants in the usual care group (Table 1). At baseline, the participants had a mean (SD) of 4.2 (1.7) diagnoses, used a median (IQR) of 8 (7-10) regular medications, 187 (42.3%) had been prescribed 1 or more PIMs, and 109 (24.7%) had fallen at least once during the previous 3 months prior to study participation. Total medication counts, PIM use, and the distribution of drug class at baseline were similar between the groups (eTables 1 and 2 in Supplement 2).

**Table 1.** Baseline Characteristics of Study Participants

Characteristic	No. (%)	
	Intervention group (n = 215)	Usual care group (n = 227)
Age, mean (SD), y	81.7 (6.9)	81.9 (7.4)
Sex		
Female	112 (52.1)	111 (48.9)
Male	103 (47.9)	116 (51.1)
BMI, mean (SD)	22.6 (4.2)	22.3 (4.7)
Smoking status		
Current or former	62 (28.8)	96 (42.3)
Never	153 (71.2)	131 (57.7)
Care level^a^		
No long-term care insurance application	99 (46.0)	105 (46.3)
Need support level	30 (14.0)	25 (11.0)
Need care level 1-2	48 (22.3)	54 (23.8)
Need care level 3-5	36 (16.7)	41 (18.1)
Missing	2 (0.9)	2 (0.9)
EQ5D-3L score, median (IQR)	0.7 (0.5-0.8)	0.7 (0.5-0.8)
Any fall during previous 3 mo	53 (24.7)	52 (23.0)
No. of diagnoses, mean (SD)	4.0 (1.7)	4.3 (1.8)
Index diseases		
Heart failure	14 (6.5)	13 (5.7)
Pneumonia	37 (17.2)	54 (23.8)
Diabetes	16 (7.4)	17 (7.5)
Ischemic stroke	11 (5.1)	7 (3.1)
Urinary tract infection	16 (7.4)	21 (9.3)
eGFR, mean (SD), mL/min/1.73 m^2^	49.5 (33.7)	50 (32.1)
No. of drugs, median (IQR)^b^	8 (7-10)	8 (7-10)
No. of PIMs, median (IQR)^c^	0 (0-1)	0 (0-1)

Abbreviations: BMI, body mass index (calculated as weight in kilograms divided by height in meters squared); EQ5D-3L, EuroQol 5 dimensions 3-levels; PIM, potentially inappropriate medication.

^a^ Care level represents the degree of caregiving needed by older adults, ranging from those who have not applied for long-term care insurance to those requiring the highest level of care in care level 5, as defined by the Japanese long-term care system.

^b^ Medications categorized as *as needed* were excluded from the medication counts.

^c^ PIM was determined based on STOPP/START criteria version 2.^20^

The mean (SD) preparation time of prescribing optimization proposals took 16.0 (7.2) minutes for both a clinician and a clinical pharmacist. The intervention team proposed a total of 737 deprescribing proposals directed to attending physicians and participants. Of these proposals, 254 (34.5%) were Anatomical Therapeutic Chemical (ATC) code A (alimentary tract and metabolism), 147 (19.9%) were ATC code N (nervous system), and 142 (19.3%) were ATC code C (cardiovascular system). Among 188 participants in the intervention group who were subjected to 1 or more recommendations for deprescribing, 153 (81.4%) accepted at least 1 proposal. The overall acceptance rate of the proposal was 68.7% (eTable 3 in Supplement 2).

**Figure 2.**
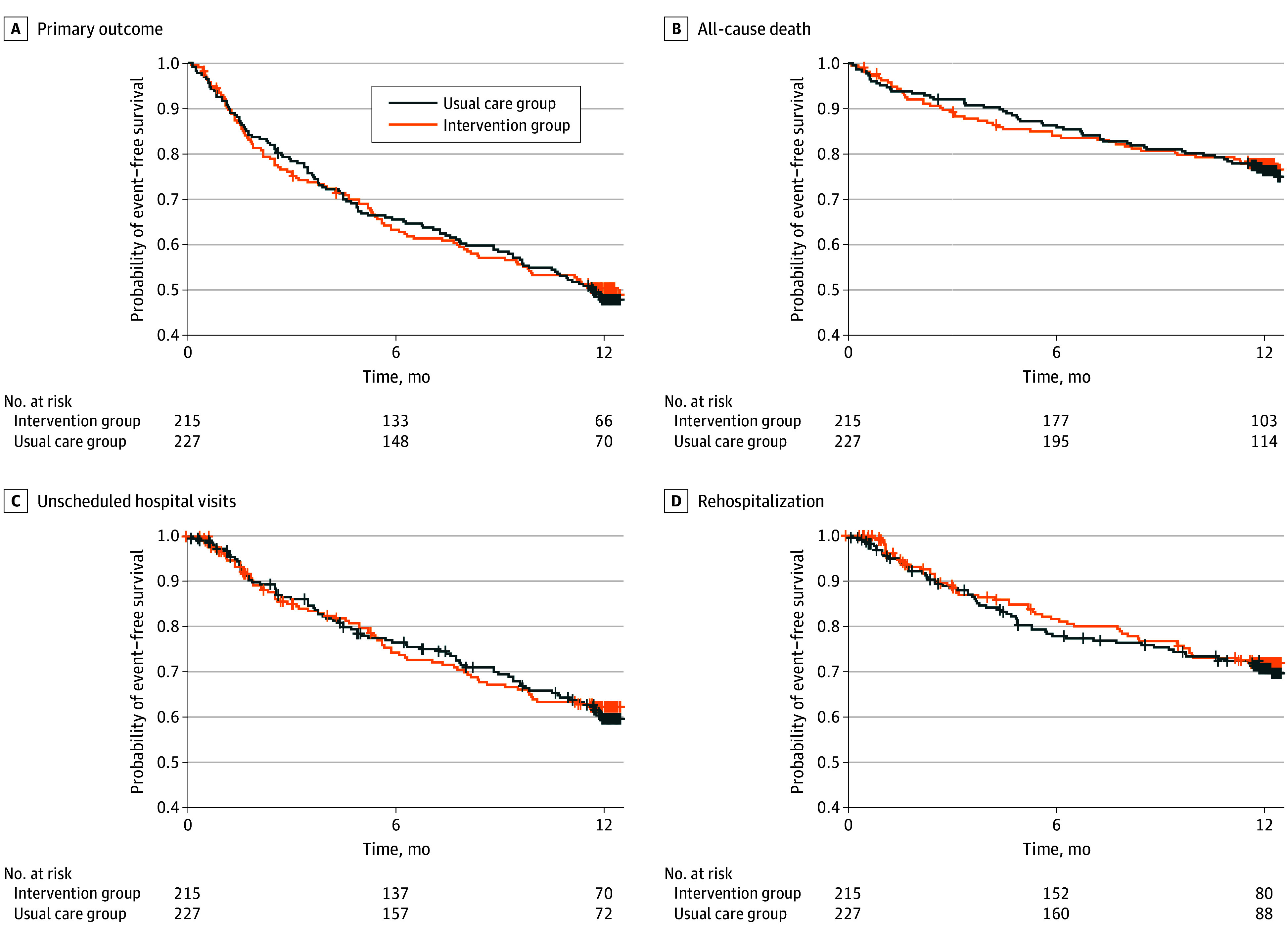
Survival Plot of Time to Event for Participants in the Intervention Group and Usual Care Group A, Primary composite outcome of all-cause death, unscheduled hospital visits, and rehospitalization. B, All-cause death. C, Unscheduled hospital visits; D, Rehospitalizations. Tick marks indicate censoring events.

The primary outcome (composite of death, unscheduled hospital visits, and rehospitalization) occurred in 106 participants (49.3%) in the intervention group and 117 participants (51.5%) in the usual care group. In an intention-to-treat analysis, there were no significant differences in the primary outcome between groups (stratified HR, 0.98 [95% CI, 0.75-1.27]; *P* = .85) (Figure 2A). In per-protocol analysis, which was restricted to 153 participants in the intervention group who received 1 or more deprescribing recommendations and accepted at least 1 proposal, the results remained nonsignificant for the primary end point (stratified HR, 1.05 [95% CI, 0.78-1.41]; *P* = .70) (eTable 4 in Supplement 2).

**Figure 3.**
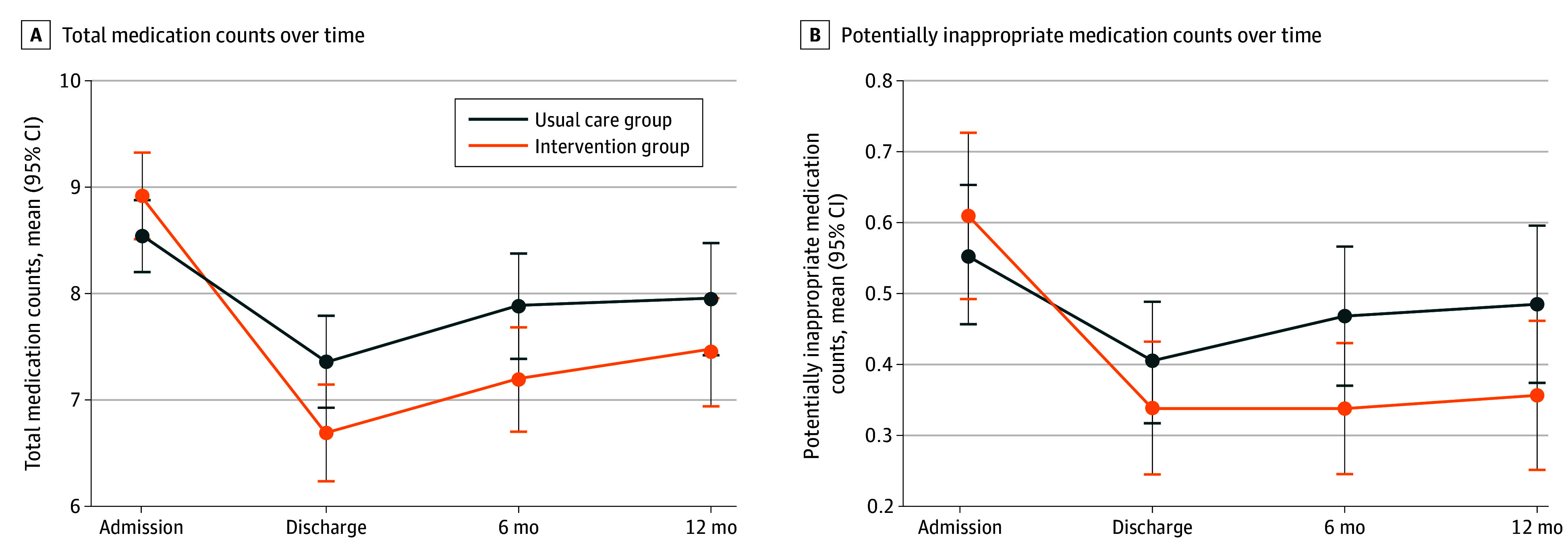
Total Medication Counts and Potentially Inappropriate Medications Over Time Each point represents the mean, and each error bar represents the 95% CI. Medications categorized as *as needed* were excluded from the medication counts.

Upon discharge, the intervention group was prescribed a mean of 0.89 (95% CI, 0.38-1.40) (*P* < .001) fewer medications than the usual care group, adjusted for baseline medication count and age-group strata. This difference in the total medication counts remained significant at 6 and 12 months, with adjusted mean differences of 0.92 (95% CI, 0.37-1.46) (*P* = .001) and 0.62 (95% CI, 0.03-1.20) (*P* = .04), respectively (Figure 3). We observed a significant decrease in the percentage of patients with 1 or more PIMs in the intervention group compared with the usual care group at discharge (26.2% vs 33.0%; adjusted odds ratio [OR], 0.56 [95% CI, 0.33-0.94]; *P* = .03), at 6 months (27.7% vs 37.5%; adjusted OR, 0.50 [95% CI, 0.29-0.86]; *P* = .01), and at 12 months (26.7% vs 37.4%; adjusted OR, 0.45 [95% CI, 0.25-0.80]; *P* = .007). (eTable 5 in Supplement 2).

Other secondary efficacy outcomes, including all-cause mortality (stratified HR, 1.00 [95% CI, 0.68-1.47]; *P* = .98), unscheduled hospital visits (stratified HR, 0.96 [95% CI, 0.70-1.32]; *P* = .80), rehospitalizations (stratified HR, 0.92 [95% CI, 0.64-1.33]; *P* = .66), level of long-term care required, health-related quality of life, and death during the initial hospitalization were similar in both groups (Figure 2B, Figure 2C, Figure 2D; Table 2). Compared with the control group, the incidence of fall and fall-related injuries in the intervention group were not statistically significantly lower (between-group difference: stratified HR, 0.86 [95% CI, 0.56-1.32]; *P* = .48) (eFigure 1 in Supplement 2).

**Table 2.** Patient-Reported Outcomes by Follow-Up and Death During Initial Hospitalization

	Intervention group		Usual care group		Adjusted difference (95% CI)	*P* value
	Total No.^a^	No. (%)	Total No.^a^	No. (%)		
Need care level 3-5^b^						
Baseline	215	36 (16.7)	227	41 (18.1)	NA	NA
6 mo	177	38 (21.5)	194	55 (28.4)	0.74 (0.40 to 1.35)^c^	.33
12 mo	162	38 (23.5)	172	50 (29.1)	0.79 (0.43 to 1.41)^c^	.42
Mean EQ5D-3L score (SD)						
Baseline	215	0.6 (0.3)	227	0.6 (0.3)	NA	NA
6 mo	177	0.8 (0.2)	194	0.8 (0.3)	0.03 (−0.01 to 0.08)^d^	.17
12 mo	162	0.8 (0.2)	172	0.8 (0.2)	0.02 (−0.03 to 0.06)^d^	.41
Adverse events	215	123 (57.2)	227	135 (59.5)	0.91 (0.63 to 1.34)^e^	.32
Serious adverse events	215	83 (38.6)	227	91 (40.1)	0.95 (0.65 to 1.39)^e^	.39
Death during initial hospitalization	215	9 (4.2)	227	9 (4.0)	1.07 (0.42 to 2.75)^e^	.45

Abbreviations: EQ5D-3L, EuroQol 5 dimensions 3-levels; NA, not applicable.

^a^ Number of participants varied as each was based on available data.

^b^ Care level represents the degree of caregiving needed by older adults, ranging from those who have not applied for long-term care insurance to those requiring the highest level of care in care level 5, as defined by the Japanese long-term care system.

^c^ Odds ratio from multivariable logistic regression model adjusted for baseline care level and age group.

^d^ Risk difference from multiple regression model adjusted for baseline EQ5D-3L score and age group.

^e^ Odds ratio from multivariable logistic regression model adjusted for age group.

Adverse events occurred in 123 participants (57.2%) in the intervention group and 135 participants (59.5%) in the control group (Table 2). Overall, adverse events were similar between groups, and there were no safety events related to the study intervention (eTable 6 in Supplement 2).

The intervention effect on primary end point did not differ in prespecified subgroup analyses both for indicator diseases (heart failure, pneumonia, diabetes, ischemic stroke, and urinary tract infection) and indicator drug classes at baseline (antiplatelets, antihypertensives, antidiabetics, and sedatives) (eFigure 2 in Supplement 2). A post hoc subgroup analysis restricted to participants in both groups with 1 or more PIMs did not show a significant difference for the primary end point (eTable 7 in Supplement 2).

## Discussion

This randomized clinical trial of older inpatients with polypharmacy investigated the efficacy of multidisciplinary team-based intervention integrating explicit and implicit criteria to promote prescribing optimization. Older inpatients receiving the intervention or usual care did not differ in all-cause death, unscheduled hospital visits, or rehospitalization. However, total medication counts were reduced in the intervention group compared with usual care, and the proportion of patients prescribed PIM was significantly lower in the intervention group than in usual care. Furthermore, no excess adverse events due to the intervention were observed.

This study demonstrated the safety of deprescribing in hospitalized patients with a high mortality rate in which more than 20% died during the 1-year follow-up. Mortality rates during the study period among previous studies ranged from 2.1% to 11.3% in the primary care setting,^25,26,27,28,29^ and from 5.6% at 90 days^30^ to 19.2% at 1 year^31^ in the inpatient setting, suggesting that our study population had a higher morbidity and mortality than previously reported studies. Our results corroborate the safety of deprescribing interventions in older inpatients with polypharmacy. The effect of our intervention on drug reduction is comparable to or exceeds that of previous studies. Although there is no consensus minimal clinically important difference for polypharmacy interventions, a meta-analysis of 25 deprescribing intervention trials reported a mean drug reduction of 0.4 drugs due to intervention.^14^ The Medication Optimization Protocol Efficacy for Geriatric Inpatients (MPEG) trial results support a potentially positive impact of deprescribing on health care costs and treatment burden; however, evidence is still limited regarding the cost-effectiveness of deprescribing in a hospital and discharge setting.^32^ Furthermore, reductions in medication counts and PIMs were maintained through 12 months, suggesting that the integration of a community-based intervention including primary care physicians and community pharmacists may have contributed to maintaining the effect of the intervention.

On the other hand, even with a design intended to overcome the limitations of existing studies, such as the 12-month long-term follow-up,^13^ our study failed to demonstrate clinical efficacy. The Kaplan-Meier survival curve of the primary outcome revealed no trend suggesting a potential difference between groups toward the latter half of the 12-month period. This is in line with previous deprescribing studies with no demonstrable effects on clinical end points.^28,33,34,35^ There are several possible explanations why the intervention did not improve the primary end point. The high mortality of the study participants, which is a competing risk for unscheduled hospital visits and rehospitalization, may have led to an underestimation of the effect of the intervention. Furthermore, in older adults with high morbidity and mortality, the effect of polypharmacy on clinical outcomes may be relatively small compared with other competing risks, making it difficult to observe explicit benefits even when deprescribing is effective. It is also possible that clinically meaningful outcome improvements were not achieved because many of the medications that were actually reduced were relatively low-risk medications (Supplement 2). From the perspective of treatment burden, the MPEG trial intervention identified medications that were potentially unnecessary and showed that reducing these low-risk medications did not increase harm. Additionally, it is possible that the single-center, open-label design may have led to contamination in delivery of the intervention, which could have attenuated the effect of intervention. However, previous cluster randomized clinical trials of deprescribing also failed to demonstrate clinical effectiveness, suggesting that the challenges in establishing the clinical effectiveness of deprescribing extend beyond study design limitations.^29,31^

Deprescribing interventions focusing on specific drug classes, such as antihypertensive or sedative medications, may not be more effective than interventions designed to reduce the total number of medications. Subgroup analyses by baseline prescription drug class (eFigure 2 in Supplement 2) in MPEG did not suggest differences in effects by indicator drug classes. This interpretation is supported by evidence suggesting that the number of medications taken is the best predictor of adverse drug events.^36^ While focusing on the number of medications as a surrogate marker is considered the cornerstone of polypharmacy interventions, future efforts should shift from simply reducing the number of drugs to focusing on patient-centered interventions to change patient attitudes and health-related behavior. Recent review articles point to the potentially greater clinical effectiveness of patient-centered interventions for deprescribing over other interventions.^12,37^ For instance, an interventional study in hospital wards, adopting multiple motivational interviews in addition to medication review, has shown a reduction in unscheduled hospital visits and rehospitalization.^24^ This effect could be attributed to active involvement of patients in polypharmacy interventions, which allow patients to be more proactive^38^ and promote subsequent behavioral change. Future research will require development and subsequent validation of replicable patient-centered, comprehensive medication review interventions across settings.

### Limitations

This study has limitations. First, the lack of blinding of participants and the deprescribing team could have potentially introduced bias into the study results. To address observer bias, research assistants were masked to treatment allocation, ensuring a more objective outcome assessment even in situations where participants needed to be fully informed about their treatment.

The single-center, open-label study design with randomization at the patient level could have influenced attending physicians’ prescribing behavior in the usual care setting. As a result, their prescription practices might not accurately reflect scenarios in clinical practice, potentially making it difficult to assess the intervention’s effectiveness and generalizability. However, to mitigate the aforementioned influence, we conducted the trial across 8 internal medicine inpatient wards, aiming to ensure a diverse range of attending physicians involved in the study.

Our study was limited to participants with an estimated duration of hospitalization of 1 week or longer. This specific eligibility criteria, important for assuring patient safety, should warrant caution when applying the results to older inpatients in general. In addition, the exclusion of as-needed medications might exclude drugs at high risk of adverse events, thus attenuating the generalizability of the current study.

The impact of the relatively high number of participants with no PIMs and the use of medication reconciliation in the control group may have biased toward null results. However, our eligibility criteria utilizing simple medication count, rather than the appropriateness of medications, may reflect situations that clinicians and pharmacists face in day-to-day clinical practice.

Additionally, our medication optimization protocol-based team discussions, while aimed at patient-centeredness, remained predominantly clinician-driven. Since our intervention utilized a similar multifaceted intervention as a previous study,^24^ we hypothesized that our intervention may have a similar effect on clinical end points. Nevertheless, it is plausible that our intervention may have lacked an important element for clinical effectiveness, such as motivational interviewing. Recent developments in personalized medicine, including research based on pharmacogenomics, have heightened interest in individualized health care. In the context of polypharmacy management, there is a growing emphasis on comprehensively understanding patients from diverse biological, psychological, and social perspectives, fostering initiatives that encourage patients to take a more active role in polypharmacy management.

## Conclusions

Our multidisciplinary team-based deprescribing intervention had no demonstrable effect on the composite end point of death, unscheduled hospital visits, or rehospitalization. However, the intervention was effective in reducing the number of medications and PIMs, with no increase in adverse events even in older inpatients with polypharmacy.

## Notes

Supplement 1.
Trial Protocol

Supplement 2.
eMethods
**eTable 1.** Top 10 Regular Medications at Baseline in the Intervention and Usual Care Group **eTable 2.** Top 10 Potentially Inappropriate Medication at Baseline in the Intervention and Usual Care Group **eTable 3.** Number of Recommendations and Acceptance Rate According to ATC Classification **eTable 4.** Per-Protocol Analysis Restricted to Participants in the Intervention Arm Who Were Subjected to One or More Recommendations for Deprescribing and Accepted the Proposal **eTable 5.** Proportion of Participants With Polypharmacy (5+ Medications), Hyper Polypharmacy (10+ Medications), and Potentially Inappropriate Medication in Both Groups **eTable 6.** Adverse Events Reported in the Intervention and Usual Care Group **eTable 7.** Post-Hoc Subgroup Analysis Restricted to Participants in Both Groups With One or More Potentially Inappropriate Medications **eFigure 1.** Kaplan-Meier Survival Plot of Time to First Fall or Fall-Related Injuries for Participants in the Intervention Group and Usual Care Group: Results From Modified Intention-to-Treat Analysis **eFigure 2.** Forest Plot of the Primary Outcome According to Subgroups

Supplement 3.
Data Sharing Statement
