# Error in Figure 1

**DOI:** 10.1001/jamanetworkopen.2024.49465

**Published:** 2024-11-12

In the Original Investigation titled “Medication Optimization Protocol Efficacy for Geriatric Inpatients: A Randomized Clinical Trial,”^1^ published July 30, 2024, the lower right box in Figure 1 listed the wrong number included in the analysis for the usual care group. It should have read, “227 Included in the analysis.” This article has been corrected.^1^

## References

[1] Ie K, Hirose M, T, . Medication optimization protocol efficacy for geriatric inpatients: a randomized clinical trial. JAMA Netw Open. 2024;7(7):e2423544. doi:10.1001/jamanetworkopen.2024.23544 PMC11289701 39078632

