# Supplementary material for: Medication Optimization Protocol Efficacy for Geriatric Inpatients: A Randomized Clinical Trial
Source: JAMA Netw Open. 2024 Jul 30;7(7):e2423544. doi: 10.1001/jamanetworkopen.2024.23544 (PMC11289701; doi:10.1001/jamanetworkopen.2024.23544)
Supplement: Supplement 1. — Trial Protocol [file jamanetwopen-e2423544-s001.pdf]

Title Page

Study protocol

Medication optimization Protocol Efficacy for Geriatric inpatients(MPEG):
A randomized clinical trial

Principal Investigator:
Kenya Ie
Department of General Internal Medicine, Kawasaki City Tama Hospital
1-30-37 Shukugawara, Tama-ku, Kawasaki, Kanagawa 214-8525, Japan
Phone number: 044-933-8111
FAX number: 044-933-8432
E-mail：iekenya0321@gmail.com

Planned duration of clinical trial:
Date of approval - December 31, 2023

Date Created/Revised:
May 17, 2023 Created ver 1.7

Notice

This document is NOT formal protocol but is the English translation version of the original protocol written in Japanese.

Revision History

Edition No.	Effective date	Author	Remarks
ver 1.0	December 10, 2018	Kenya Ie	Protocol approved by the St. Marianna University School of Medicine Institutional Ethical Committee (No. 4129).
ver 1.1	May 13, 2019	Kenya Ie	First revision approved.<br>-Clarification of wording in protocol and addition/removal of research team members.
ver 1.2	October 9, 2019	Kenya Ie	Second revision approved.<br>-It was found that preparing a nursing summary at discharge with detailed deprescribing information was difficult due to time constraints. Since the intervention team already prepared a drug information report as part of the planned intervention, it was deemed unnecessary to prepare a nursing summary for all participants unless required as a part of the usual care process.
ver 1.3	September 8, 2020	Kenya Ie	Third revision approved.<br>-CTCAE version update<br>- Change in research team member's affiliation
ver 1.4	January 12, 2021	Kenya Ie	Fourth revision approved.<br>-Extension of the study period due to the delay in study enrollment, partly because of the COVID-19 pandemic.
ver 1.5	June 22, 2021	Kenya Ie	Fifth revision approved.<br>-Extension of the study period due to the delay in study enrollment, partly because of the COVID-19 pandemic.<br>-Change in research team member's job title
ver 1.6	December 2, 2021	Kenya Ie	Sixth revision approved.<br>-Extension of the study period due to the delay in study enrollment, partly because of the COVID-19 pandemic.
ver 1.7	June 21,2023	Kenya Ie	Seventh revision approved.<br>-Extension of the study period due to delays in data entry.<br>-Change in research team members’ job title and affiliation.

Table of contents

1. *Background* *1*
2. *Necessity and objective of the study* *1*
3. *Overview of the intervention* *2*
4. *Participants* *2*
5. *Informed consent* *2*
6. *Study methods* *3*
7. *Endpoints* *5*
8. *Examination and evaluation* *5*
9. *Criteria for study discontinuation* *8*
10. *Handling of adverse events* *8*
11. *Reporting deviations from the protocol* *9*
12. *Termination, Discontinuation or Suspension of the Study* *9*
13. *Duration of the study* *10*
14. *Data aggregation and statistical analysis methods* *10*
15. *Sample size calculation and its rationale* *11*
16. *Consideration for human rights, safety and disadvantage of study participants* *11*
17. *Cost burden for study participants* *11*
18. *Indemnification and clinical trial participants insurance* *11*
19. *Compliance with the Declaration of Helsinki* *12*
20. *Data management* *12*
21. *Publication of study results* *12*
22. *Central monitoring and audit* *12*
23. *Organization* *13*
24. *References* *15*

Main text

1. Background

Polypharmacy is a growing social problem that increases hospitalizations and deaths among the elderly, and also leads to higher healthcare costs. As a countermeasure, a list of potentially inappropriate medications that may lead to adverse drug events has been widely used. One list that has been particularly widely disseminated is the Screening Tool of Older Persons' Prescriptions (STOPP).[1] One feature of this list is that it can be used in conjunction with the Screening Tool to Alert to Right Treatment (START) criteria, which summarize recommended treatments that are beneficial to the elderly, along with the STOPP criteria, which summarize potentially inappropriate prescribing in the elderly.[1] The STOPP/START list has been widely used in the United States and Europe. A recent systematic review of randomized controlled trials of deprescribing interventions using STOPP/START showed that STOPP/START interventions were associated with fewer falls, shorter hospital stays, fewer emergency room visits, and lower healthcare costs. However, to date, no improvements in more important outcomes, such as death or rehospitalization, have been demonstrated with the intervention.[2] Limitations of the list of potentially inappropriate medications include the large number of adverse events caused by drugs not included on the list, as well as the possibility that there is both "appropriate polypharmacy" and "inappropriate polypharmacy".

In response to these limitations, "deprescribing protocol",[3] in which the appropriateness of individual prescription medications for each patient is reviewed based on certain criteria, have gained attention. Although protocol-based interventions have been shown to reduce the number of prescribed medications,[4] it has yet to be proven whether these interventions improve important clinical outcomes such as death, hospitalization, and falls.[5,6,7] However, it has been suggested that most recent studies have had short follow-up periods of less than one year and may not have detected a true effect.[5] Thus, while polypharmacy intervention is recognized as an important topic worldwide, methodological problems with existing studies have also been highlighted.

2. Necessity and objective of the study

In Japan, healthcare policy toward deprescribing through the Comprehensive Drug Evaluation and Adjustment Program started in 2016, and there is a social need to verify whether deprescribing is effective and worth the effort and cost. Furthermore, as mentioned above, if the true efficacy of polypharmacy intervention can be proven in clinical trials addressing methodological limitations of existing research, it is expected to be a globally valuable evidence that will promote the implementation of deprescribing interventions.

This study will examine the effect of multidisciplinary team-based medication optimization on survival, unscheduled hospital visits, and rehospitalization in elderly inpatients with polypharmacy in a single-center, parallel-group comparative trial.

3. Overview of the intervention

A prescription optimization support team consisting of physicians, pharmacists, and nurses reviews the prescriptions of internal medicine inpatients in the intervention group based on STOPP/START and the prescription optimization protocol specifically designed for this study, and makes prescription adjustment proposals after discussion within the team. The name of the drug suggested to be added, reduced, or discontinued, the reason for the change, and the precautions to be taken after the prescription change are generated by the clinical decision support system and provided to the attending physician of the participant. The implementation of prescription changes is left to the discretion of the attending physician as part of usual care.

4. Participants

(1) Inclusion Criteria:

Patients who meet all of the following criteria will be considered eligible.

1. Medical inpatients admitted to the internal medicine departments of Kawasaki Municipal Tama Hospital (general internal medicine, gastroenterology&hepatology, cardiology, pulmonology, endocrinology, nephrology, and neurology) at Kawasaki Municipal Tama Hospital;
2. Aged 65 years or older;
3. Taking five or more regularly prescribed medications;
4. Predicted length of hospital stay after admission: 1 week or longer;
5. Patients who are deemed eligible to take the drug orally by their attending physician;
6. Patients who have been fully informed of the study and who have given their or their surrogate's free and voluntary written consent based on a thorough understanding of the study.

(2) Exclusion Criteria:

Patients will not be included in the study if the following apply.

1. Attending physicians disagreeing to study participation;
2. Life expectancy of less than 1 month based on their attending physician’s clinical judgement.

5. Informed consent

Prior to participation in this study, all participants will be informed orally and written consent will be obtained. However, if the investigator determines that the participant himself/herself is incapable of making a decision, consent will be obtained from a participant’s next of kin (NoK). In this study, a NoK is defined as a person who is justified in giving consent on behalf of the participant based on the relationship and psychological

connection with the participant. In this case, if the NoK is unable to come to the hospital on the same day, he/she will participate in the study with verbal consent by telephone and obtain written consent at a later date. When there is a change in the research protocol that may affect the participant's consent, their intention to participate in the study should be promptly confirmed, and the consent document, should be revised with the prior approval of the President to obtain the participant's consent again.

6. Study methods

（1） Design

Single-center open-label prospective randomized controlled trial

（2） Trial outline（Figure 1）

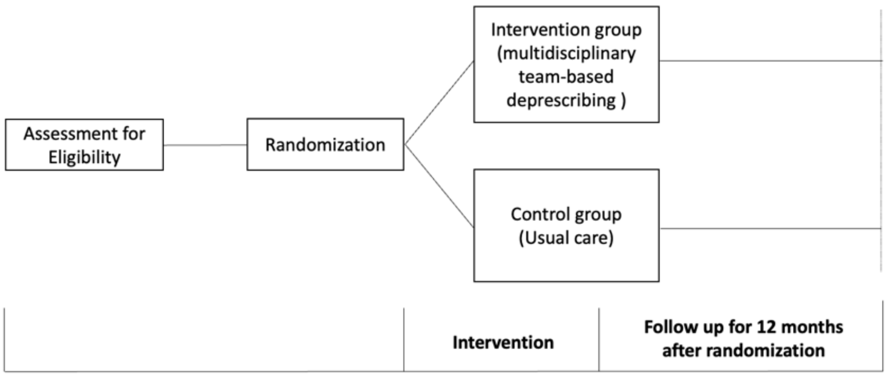

（3） Expected duration of participant's enrollment in the study

Participant will enroll in the study from the time of allocation until the completion of 12-month follow-up. During this time, participants will be followed up by periodic telephone interviews.

（4） Intervention

The prescription optimization scheme for the intervention group is shown in Figure 2. For subjects assigned to the intervention group after consent is obtained, the prescription optimization support team collects and reviews information and proposes a prescription adjustment plan to the team in charge within 2 days of assignment. Specifically, drug names, blood test data, disease names, etc. are entered into the computerized prescription optimization support system, and the system automatically creates a prescription optimization draft based on STOPP/START. The Prescribing Optimization Support Team, consisting of a physician, pharmacist, and nurse, then conducts a drug review of the subject based on the Prescribing Optimization Protocol (Figure 3) and develops a Prescribing Adjustment Plan. Prescription suggestions (addition, reduction,

discontinuation, etc.), their rationale, and precautions after prescription changes are documented and provided in writing to the physician in charge, leaving the final prescription details to the judgment of the physician in charge. After a change in prescription, if the ward nurse, pharmacist, or physician in charge provides information on any changes that may be related to the prescription, the Prescribing Optimization Support Team will respond as appropriate. At the time of discharge, the Prescription Optimization Support Team will prepare a drug information report after confirming the participant's condition, and provide it to the primary care physician.

Figure 2.

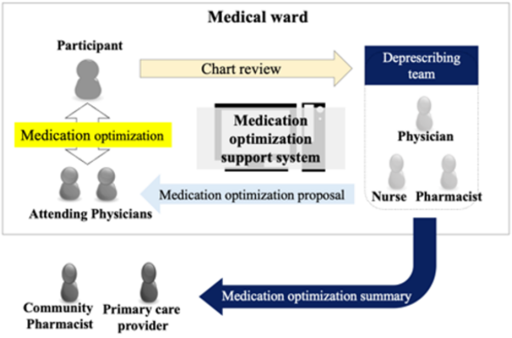

Figure 3.

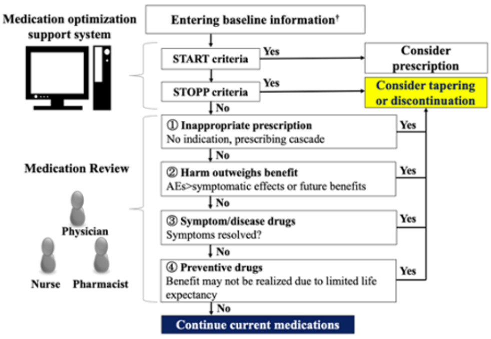

（5） Case registration and allocation methods

【Allocation methods】

Patients will be assigned to the deprescribing intervention group and usual care group in a 1:1 ratio by a stratified block randomization method. The allocation factors will be age groups (65-74 years, 75-84 years, and 85 years and older).

【Case registration procedure】

The following procedure will be used to enroll cases by the person in charge of the research or a research collaborator.

(1) Obtain written consent.

(2) Enter the date consent was obtained and the necessary information to correspond the subject and subject identification code on the subject identification code list kept by the principal investigator.

(3) Register the case registration form with the subject identification code in eACReSS.

(4) Receive confirmation of eligibility and a registration confirmation form with the subject registration number and the group to which the subject has been allocated.

(5) Promptly report to the principal investigator any withdrawal, discontinuation, or dropout.

7. Endpoints

（１） Primary endpoint

- Time to rehospitalization, unscheduled visit, or all-cause death

（２） Secondary endpoints

- All-cause death
- Unscheduled hospital visit
- Rehospitalization
- All-cause deaths during initial hospitalization
- Fall
- Health-related quality of life score (EQ-5D Japanese version)
- Level of long-term care required
- Number of regularly prescribed medications
- Number of potentially inappropriate medications listed in the STOPP criteria version 2[1].
- Human and time costs of interventions

（３） Safety endpoints

- Adverse events associated with the intervention will be recorded according to CTCAE version 5.0.

8. Examination and evaluation

(1) Participant background

Subject identification code, initial, gender, date of birth, date of hospital admission, race, height at admission, weight at admission, BMI at admission, vital signs at admission (blood pressure, pulse rate, temperature), comorbidities, past medical history, smoking status, history of falls within 3 months prior to enrollment, level of care required, prescription drug information, laboratory results at admission (serum creatinine level, serum sodium level, serum potassium level, eGFR, pO2, pCO2), current medical history (name of diagnosis) will be obtained at the time of enrollment.

(2) Intervention Items

Allocation group.

(3) Endpoints

＜Primary endpoints＞

All-cause deaths, unscheduled visits, and rehospitalizations up to 12 months after allocation will be confirmed

by telephone survey every 2 months, including specific dates, referring to the entries in the follow-up diary provided in advance. A hospital transfer is considered to be a continued hospitalization, and a rehospitalization is defined as "hospitalization due to any new event”. If necessary, we will request information from the patient's primary care institution, primary care pharmacy, home nursing station, after making a request for research cooperation in advance.

＜Secondary endpoints＞

After 6 months and 12 months of enrollment, the researcher or research collaborator will contact the participant via telephone and interview about the following information. If it is deemed difficult to interview the participants themselves, a NoK will be asked the same information. Information on medications will be recorded at the time of enrollment and hospital discharge, and inquiries will be made by telephone or fax to the community pharmacy at 6 months and 12 months.

1. Symptoms: To be confirmed by interview.
2. Adverse events: Detailed of events, date of onset/disappearance, degree, treatment required, outcome, severity assessment, and relevance to the intervention will be reported using case report form according to CTCAE version 5.0.
3. Number of medications: Descriptive statistics of the number of medications taken at admission, discharge, and 6 and 12 months after study enrollment and the proportion of drug names.
4. Number of potentially inappropriate prescriptions: Descriptive statistics of the number of potentially inappropriate prescriptions and the proportion of potentially inappropriate drug names. Potentially inappropriate prescriptions are defined as medications that meet STOPP criteria version 2[1].
5. Health-related quality of life: Measured at baseline, 6 months, and 12 months using the EQ-5D Japanese version. At baseline, a face-to-face survey will be conducted by the researcher, and thereafter, interviews will be conducted via telephone.
6. Level of long-term care required: To be ascertained by interviewing the patient or NoK.
7. Falls and injuries due to falls

Table. Timetable of the MPEG trial

	Baseline assessment	Enrollment	Follow-up 1	Follow-up 2	Follow-up 3	Follow-up 4	Follow-up 5	Follow-up 6
**Time point (week)**	0	0	8 ± 2	16 ± 2	24 ± 2	32 ± 2	40 ± 2	48 ± 2
**Enrollment**								
Informed consent	x							
Sociodemographic characteristics	x							
Allocation		x						
**Intervention**		x						
**Assessments**								
Subjective symptoms	x	x	x	x	x	x	x	x
Adverse events			x	x	x	x	x	x
Vital signs	x							
Height and weight	x							
Unscheduled visits			x	x	x	x	x	x
Hospital readmission			x	x	x	x	x	x
Injury due to falls			x	x	x	x	x	x
Laboratory tests (eGFR, serum sodium level, and serum potassium level)	x							
Number of prescribed medications	x				x			x

Number of prescribed potentially inappropriate medications*	x				x			x
EQ5D-3L		x			x			x
Level of long-term care required	x				x			x

*The number of prescribed potentially inappropriate medications listed in the STOPP/START criteria version 2[1].

9. Criteria for study discontinuation

(1) When a subject declines to participate in the study or withdraws consent

(2) When eligibility is found to be unmet after enrollment

(3) When it is difficult to continue the study due to exacerbation of complications

(4) When adverse events make it difficult to continue the study

(5) When the entire study is terminated

(6) The investigator deems it appropriate to terminate the study for other reasons.

If the investigator determines that the study cannot be continued for one or more of the above reasons, the intervention will be stopped immediately and the date of discontinuation or dropout, reason for discontinuation or dropout, and progress will be noted in the medical record, and necessary tests will be performed at the time of discontinuation or dropout to evaluate efficacy and safety. If the intervention is discontinued due to an adverse event, the patient should be followed up as much as possible until the patient recovers to the original condition.

10. Handling of adverse events

（1） Treatment of participants with adverse events

The medication adjustment proposal in the intervention group in this study is not mandatory, and actual prescription decision and follow-up will be made at the discretion of the attending physician. Therefore, the risk of adverse events in the intervention group is not expected to deviate significantly from usual care.

However, in the unlikely event of an adverse event associated with the intervention, the principal investigator or study personnel will take appropriate action immediately.

（2） Reporting of serious adverse events

Serious adverse events in this study are defined as follows.

1) Death or threat of death
2) Hospitalization or prolonged hospitalization
3) Disability or risk leading to disability
4) Acquired or congenital disease or abnormality

All serious adverse events during the study period will be reported by the principal investigator to the president (Chairman of the Institutional Ethical Committee).

11. Reporting deviations from the protocol

The investigator or principal investigator may deviate from or change the study protocol for compelling reasons, such as emergency avoidance, before obtaining prior approval from the President (Institutional Ethical Committee). In such cases, the investigator or principal investigator shall record all details and reasons for the deviation or change, and if a revision of the study protocol is necessary, the proposed revision will be promptly submitted to the Institutional Ethical Committee for deliberation and approval by the President.

12. Termination, Discontinuation or Suspension of the Study

(1) Termination of the study

At the end of the study, the principal investigator shall promptly submit a study termination report to the President.

(2) Discontinuation or interruption of the study

The principal investigator will consider whether or not to continue the study in the following cases

1. When it is judged to be difficult to achieve the planned number of enrollment due to difficulties in recruitment.
2. When the Institutional Ethical Committee requests changes in the protocol, and it is judged to be difficult to accept such changes.
3. When the Institutional Ethical Committee recommends or instructs to discontinue the study, the study will be terminated.

When a decision is made to discontinue or suspend a study, the decision will be promptly reported in writing

to the President, together with the reason for the discontinuance or suspension.

13. Duration of the study

The enrollment period for the study is planned to be 3 years and 3 months from the Institutional Ethical Committee approval, but may be extended or terminated earlier depending on the status of enrollment. The follow-up period for each subject will be one year.

14. Data aggregation and statistical analysis methods

Data will be stored in eACReSS, and data will be compiled after data cleaning by the data center prior to statistical analysis.

(1) Analysis set

The analysis of the primary and secondary endpoints will include all study participants enrolled in the study. If it is found that the eligibility criteria are not met after the start of the study, they will be excluded from the analysis. The analysis of the safety endpoints will be performed on all participants enrolled in the study.

(2) Statistical analysis methods

Participants who can no longer be contacted during the follow-up will be treated as censored when they were last contacted. Discontinuation of the study participation will not be considered a termination, thus observation will continue.

For the primary endpoint analysis, a survival function will be estimated for each group using the Kaplan-Meier method, which will be used to estimate survival rates and 95% confidence intervals at 6 and 12 months. Group comparisons will be made using stratified log-rank tests (two-sided) with stratified age groups (65-74, 75-84, 85+) as the strata. The significance level is set at 0.05. The baseline hazard will be stratified based on age group strata, and the hazard ratio and its 95% confidence interval will be calculated for the intervention group compared to the usual care using a Cox regression model with the time until rehospitalization, unscheduled hospital visit, or all-cause death as the outcome variable and the allocation group as the explanatory variable.

The same analysis as above will be performed for each of the time to rehospitalization, unscheduled visits, and all-cause mortality, which comprise the primary composite endpoint. The planned statistical tests are unadjusted for the multiplicity.

Subgroup analysis of the primary endpoint will be performed for each index disease (heart failure, pneumonia, diabetes, stroke, urinary tract infection) and drug class (antiplatelet/anticoagulant,

antihypertensive, antidiabetics, sedatives). The 12-month survival rate will be estimated for each subgroup by group and evaluated to see if the difference between groups is in the same direction as the overall analysis.

Details of the analysis methods for secondary endpoints and safety endpoints will be defined in a separate statistical analysis plan.

15. Sample size calculation and its rationale

Based on previous studies, we estimate the event rate at 12 months for the primary endpoint to be 30% for the intervention group and 40% for the control group. Assuming equally spaced enrollment and allocation, a total of 423 subjects would be needed for the analysis to have a power of 0.80 with a two-sided test at a significance level of 0.05. We estimate 15-20% exclusion from the analysis and plan to enroll a total of 500 subjects (250 in each group) over the 2-year period.

16. Consideration for human rights, safety and disadvantage of study participants

Candidates for study participation will be provided with sufficient information prior to study enrollment, and only those who have given written consent to participate will be included in the study. We plan to fully explain that: participation in the study is voluntary and not compulsory; no disadvantage will be incurred if consent is not given; consent can be withdrawn at any time even if requested; in such cases, the clinical information collected will be destroyed and not used for research; data is personal information and will be strictly managed; and the data will be kept in a locked cabinet, separated from personal identifying information (e.g. name, date of birth, patient ID). Personal information including test values will be anonymized so that individuals cannot be identified. Anonymized information collected in this study may be used for secondary analyses in the future with the approval of the Institutional Ethical Committee, and this will be clearly stated and fully explained in the explanation and consent document.

17. Cost burden for study participants

Since this study will be conducted as part of usual patient care, participation in the study will not result in an increase in cost burden for the subjects. Participants will receive rewards worth 500 yen for the time they spend during follow-up telephone interviews.

18. Indemnification and clinical trial participants insurance

Since this study will be conducted as part of usual patient care, there is no plan to provide indemnification for

adverse events during study participation. Therefore, no clinical trial participants insurance is required for conduct of this study.

19. Compliance with the Declaration of Helsinki

This study will be conducted in compliance with the Declaration of Helsinki (revised in 2013), "Ethical Guidelines for Medical Research Involving Human Subjects," "Nuremberg Code," and "Personal Information Protection Law.

20. Data management

The data collected in this study will be managed by the personal information manager of the Department of General Internal Medicine, Kawasaki Municipal Tama Hospital, of which the University is the designated administrator, in the investigator's research space in a manner that complies with the Personal Information Protection Law. The data will be kept until five years after the study is completed. The principal investigator will retain essential documents related to the conduct of the study (e.g. notification documents from the President, copies of various application forms and reports, subject identification code lists, consent forms, copies of case report forms, and other documents or records necessary to ensure the reliability of the data) and destroy them five years after the study is completed.

21. Publication of study results

This study will be registered in the UMIN Clinical Trials Registry System (UMIN-CTR) before the study begins. The results of this study will be reported by the principal investigator as the first author at a conference and in a peer-reviewed journal after completion of the study, which is expected to be in FY2023.

22. Central monitoring and audit

Central monitoring confirms from the input data that the investigator is appropriately conducting the study in accordance with the study protocol. In principle, central monitoring is conducted at least once a year, and the results are summarized in a monitoring report, which is submitted to the President of the institution. Although on-site monitoring and audits are not planned, direct on-site monitoring and audits will be considered depending on the results of the monitoring report.

23. Organization

【Research team】（○：Principal investigator）

- Kenya Ie ○

  Associate Professor, Department of General Internal Medicine, Kawasaki Municipal Tama Hospital (St. Marianna University School of Medicine)

  +8144-933-8111（PHS 72-8151）

- Masanori Hirose

  Assistant Professor, Department of General Internal Medicine, St. Marianna University School of Medicine

- Ayako Tsuboya

  Chief, Department of Pharmacy, Kawasaki Municipal Tama Hospital (St. Marianna University School of Medicine)

- Hiroshi Matsumoto

  Chief, Department of Pharmacy, Kawasaki Municipal Tama Hospital (St. Marianna University School of Medicine)

- Eisuke Inoue

  Professor, Showa University Research Promotion Center

- Masaki Takahashi

  Assistant Professor, Division of Medical Informatics, St. Marianna University School of Medicine

- Eiko Komiya

  Chief, Department of Pharmacy, Kawasaki Municipal Tama Hospital (St. Marianna University School of Medicine)

- Yuka Ito

  Deputy Director, Department of Pharmacy, Kawasaki Municipal Tama Hospital (St. Marianna Medical University)

- Hikari Hashi

  Chief, Department of Pharmacy, Kawasaki Municipal Tama Hospital (St. Marianna University School of Medicine)

- Tomoya Tsuchida

  Lecturer, Department of General Medicine, St. Marianna University School of Medicine

- Iori Motohashi

  Assistant Professor, Department of General Medicine, Kawasaki Municipal Tama Hospital (St. Marianna University School of Medicine)

- Shota Asamizu

  Chief, Department of Nursing, Kawasaki Municipal Tama Hospital (St. Marianna University School of

Medicine)

- Takahide Sadakata

  Deputy Chief, Department of Nursing, Kawasaki Municipal Tama Hospital (St. Marianna University School of Medicine)

- Chisato Sekikawa

  Deputy Chief, Department of Nursing, Kawasaki Municipal Tama Hospital (St. Marianna University School of Medicine)

- Takahide Matsuda

  Specially Appointed Professor, Department of General Medicine, St. Marianna University School of Medicine

【Personal Information Manager】

- Tsubasa Sakai

Assistant Professor, Department of General Internal Medicine

Kawasaki Municipal Tama Hospital (St. Marianna University School of Medicine)

044-933-8111（PHS 8054）

【Research Office】

1-30-37 Shukugawara, Tama-ku, Kawasaki, Kanagawa 214-8525, Japan

Contact person: Miyuki Kondo

Phone: 044-933-8111 (ext. 2226) FAX: 044-930-5181

E-mail: s-tsuchiya@marianna-u.ac.jp

【Patient Registration and Allocation Center】

Patient registration and allocation in this study will be conducted using eACReSS, an electronic data capture system that can be used from personal computer restricted to internet access within campus. The eACReSS system will be managed by Masaki Takahashi, Department of Medical Informatics, St. Marianna University School of Medicine.

【Data center】

2-16-1 Sugao, Miyamae-ku, Kawasaki, Kanagawa 216-8511, Japan

Clinical Research Data Center, St. Marianna University School of Medicine

Phone: 044-977-8111 (ext. 6191) Fax: 044-977-8166

E-mail: datacenter@marianna-u.ac.jp

【Statistical Analysis Manager】
Showa University Building 1, 3F, 1-5-8 Hatanodai, Shinagawa-ku, Tokyo 142-8555, Japan
Professor Eisuke Inoue, Showa University Research Promotion Center
Phone: 03-3784-8000 FAX: 03-3784-8012
E-mail: eisuke.inoue@med.showa-u.ac.jp

【Statistical Analyst】
2-16-1 Sugao, Miyamae-ku, Kawasaki, Kanagawa 216-8511, Japan
Assistant Professor Masaki Takahashi, Division of Medical Informatics, St. Marianna University School of Medicine
Phone: +81-44-977-8111 FAX: +81-44-976-3239
E-mail: masaki.takahashi@marianna-u.ac.jp

【Research Funding and Conflict of Interest】
This study is supported by JSPS Grant-in-Aid for Scientific Research JP18K15434.
The principal investigators and researchers do not have any conflicts of interest to declare in this study.

24. References

1. O'Mahony D, O'Sullivan D, Byrne S, O'Connor MN, Ryan C, Gallagher P. STOPP/START criteria for potentially inappropriate prescribing in older people: version 2. Age and Ageing. 2015;44(2):213-218.
2. Hill-Taylor B, Walsh KA, Stewart S, et al. Effectiveness of the STOPP/START (Screening Tool of Older Persons' potentially inappropriate Prescriptions/Screening Tool to Alert doctors to the Right Treatment) criteria: systematic review and meta-analysis of randomized controlled studies. J Clin Pharm Ther 2016;41:158–69.
3. Scott IA, Hilmer SN, Reeve E, et al. Reducing Inappropriate Polypharmacy. *JAMA Intern Med*. 2015;175(5):827-834.
4. Potter K, Flicker L, Page A, Etherton-beer C. Deprescribing in Frail Older People: A Randomised Controlled Trial. *PLoS One*. 2016:in press.
5. Christensen M, Lundh A. Medication review in hospitalised patients to reduce morbidity and mortality (Review). *Cochrane Libr*. 2016;(2):80.
6. Johansson T, Abuzahra ME, Keller S, et al. Impact of strategies to reduce polypharmacy on clinically relevant endpoints: a systematic review and meta-analysis. *Br J Clin Pharmacol*. 2016:532-548.
7. Patterson S. Interventions to improve the appropriate use of polypharmacy for older people (Review) www.cochranelibrary.com. *Cochrane Database Syst Rev*. 2014;(10).

Medication optimization Protocol Efficacy for Geriatric inpatients(MPEG):
A randomized clinical trial

Statistical Analysis Plan

Ver.1.2

Author: Masaki Takahashi

First Completion Date: January 6, 2023
Last update: October 25, 2023

Responsible for statistical analysis: Eisuke Inoue
Date of Approval: October 20, 2023

Notice

This document is NOT formal SAP but is the English translation version of the original SAP written in Japanese.

Revision History

Edition No.	Creation Date	Author	Remarks
1.0	January 6, 2023	Masaki Takahashi	First edition enacted
1.1	May 9, 2023	Masaki Takahashi	10. Addition and modification of safety analysis items
1.2	October 25, 2023	Masaki Takahashi	After the analysis was conducted, it was found that the calculation method of hazard ratios differed from that in the study protocol, so a section on stratified hazard ratios was added.

Table of Contents

1. Objective 1
2. Outline of the Study 1
  2.1 Summary of Study Protocol 1
  2.2 Number of participants required 3
3. Analysis set 3
4. Data handling 3
  4.1 Handling of missing data and outlier 3
  4.2 Allowance range of time-specific measurements 4
  4.3 Definition of endpoints 4
5. Definition related to statistical analysis 4
  5.1 Definition related to statistical analysis methods 4
  5.2 Number of digits displayed 5
  5.3 Software used for analysis 5
6. Classification of participants 5
  6.1 Classification of participants 5
  6.2 Discontinuation or dropout from the study 5
7. Participant background 5
8. Primary endpoint 6
  8.1 Time to rehospitalization, unscheduled visit, or all-cause death 6
    8.1.1 Estimation of Survival Functions 6
    8.1.2 Event rate 6
    8.1.3 Between-group comparison 6
    8.1.4 Hazard ratio 6
    8.1.5 Stratified hazard ratio 6
  8.2 Time to rehospitalization 6
  8.3 Time to unscheduled hospital visit 7
  8.4 Time to all-cause death 7
  8.5 Subgroup analysis according to index diseases 7
    8.5.1 Time to first occurrence of any of rehospitalization, unscheduled visit, or all-cause death 7
    8.5.2 Time to rehospitalization 7
    8.5.3 Time to unscheduled hospital visit 7
    8.5.4 Time to all-cause death 7
  8.6 Subgroup analysis according to index drug class 8
  8.7 List of variables related to primary endpoint 8

9. Secondary endpoints 8
9.1 Death during initial hospitalization 8
9.2 Falls and fall-related injuries 8
9.3 Unscheduled hospital visit 8
9.4 Health-related quality of life score (EQ-5D Japanese version) 9
9.5 Level of long-term care required 9
9.6 Number of regularly prescribed medications and drug name 9
9.7 Number of potentially inappropriate medications and drug name 9
9.8 Human and time costs of interventions 9
10. Safety 9
10.1 Adverse events 9
10.2 Adverse events according to event type 9
10.3 Adverse events according to event type and severity 9
10.4 List of adverse events 10
10.5 Adverse events associated with the primary endpoint 10
10.6 Adverse events associated with the primary endpoint according to event type 10
10.7 Adverse events associated with the primary endpoint according to event type and severity 10
10.8 Events associated with primary endpoints 10
11. Reference 10

1. Objective

The purpose of this document, "Medication optimization Protocol Efficacy for Geriatric inpatients(MPEG): A randomized clinical trial Statistical Analysis Protocol" (hereafter referred to as the "Analysis Protocol"), is to describe the details of the statistical analyses in the "Medication optimization Protocol Efficacy for Geriatric inpatients(MPEG): A randomized clinical trial" (hereafter referred to as the "Study").

2. Outline of the Study

2.1 Summary of Study Protocol

Objective	This study will examine the effect of a multidisciplinary team-based intervention utilizing complementary STOPP/START and prescribing optimization protocols on death, rehospitalization, and unscheduled visits in elderly hospitalized patients in a single-center, parallel-group comparative study.
Design	・Single-center open-label prospective randomized controlled trial<br>・Trial outline<br>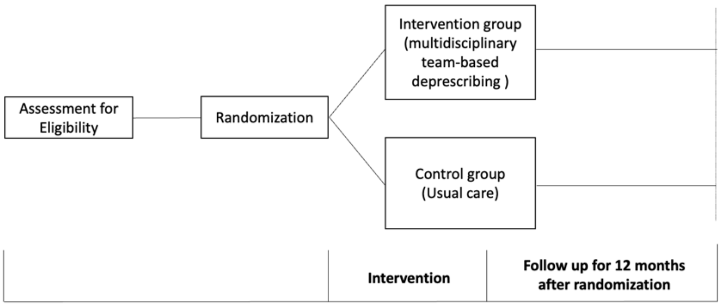<br>・Expected duration of subject's participation in the study<br>Subjects will participate in the study from the time of allocation until the 12-month point. During this time, subjects will be followed up by periodic telephone interviews.
Population	Inpatients 65 years and older<br>**<u>Inclusion criteria</u>**<br>1) Medical inpatients admitted to Kawasaki Municipal Tama Hospital;<br>2) Aged 65 years or older;<br>3) Taking five or more regularly prescribed medications*;<br>4) Predicted length of hospital stay after admission: 1 week or longer;<br>5) Patients who are deemed eligible to take the drug orally by their attending physician;<br>6) Patients who have been fully informed of the study and who have given their or their surrogate's free and voluntary written consent based on a thorough understanding of the study.<br>**<u>Exclusion criteria</u>**<br>1) Attending physicians disagreeing to study participation;<br>2) Life expectancy of less than 1 month based on their attending physician's clinical judgement.

<table>
<tr><td>Intervention</td><td>The prescription optimization scheme for the intervention group is shown in Figure 1. For subjects assigned to the intervention group after consent is obtained, the prescription optimization support team collects and reviews information and proposes a prescription adjustment plan to the team in charge within 2 days of assignment. Specifically, drug names, blood test data, disease names, etc. are entered into the computerized prescription optimization support system, and the system automatically creates a prescription optimization draft based on STOPP/START. The Prescribing Optimization Support Team, consisting of a physician, pharmacist, and nurse, then conducts a drug review of the subject based on the Prescribing Optimization Protocol (Figure 2) and develops a Prescribing Adjustment Plan. Prescription suggestions (addition, reduction, discontinuation, etc.), their rationale, and precautions after prescription changes are documented and provided in writing to the physician in charge, leaving the final prescription details to the judgment of the physician in charge. After a change in prescription, if the ward nurse, pharmacist, or physician in charge provides information on any changes that may be related to the prescription, the Prescribing Optimization Support Team will respond as appropriate. At the time of discharge, the Prescription Optimization Support Team will prepare a drug information report after confirming the participant's condition, and provide it to the primary care physician.
Figure 1.
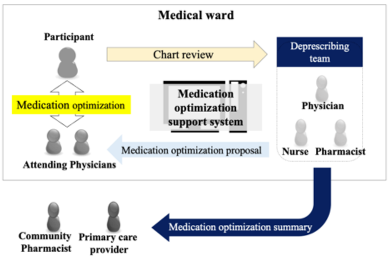

Figure 2.
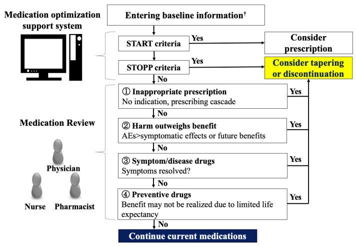
</td></tr>
<tr><td>Combination drug therapy</td><td>N/A</td></tr>
<tr><td>Prohibited drug during the study</td><td>N/A</td></tr>
<tr><td>Supportive care</td><td>N/A</td></tr>
<tr><td>Deferred treatment</td><td>N/A</td></tr>
</table>

Endpoints	【Primary endpoint】<br>Time to rehospitalization, unscheduled visit, or all-cause death<br>【Secondary endpoints】<br>• Deaths during initial hospitalization<br>• Fall<br>• Unscheduled hospital visit<br>• Health-related quality of life score (EQ-5D Japanese version)<br>• Level of long-term care required<br>• Number of regularly prescribed medications<br>• Number of potentially inappropriate medications listed in the STOPP criteria version<br>• Human and time costs of interventions<br>【Safety endpoints】<br>Adverse events associated with the intervention
Required number of subjects	500
Study period	Date of approval - June 30, 2023

2.2 Number of participants required

Based on previous studies, we estimate the event rate at 12 months for the primary endpoint to be 30% for the intervention group and 40% for the control group. Assuming equally spaced enrollment and allocation, a total of 423 subjects would be needed for the analysis to have a power of 0.80 with a two-sided test at a significance level of 0.05. We estimate 15-20% exclusion from the analysis and plan to enroll a total of 500 subjects (250 in each group) over the 2-year period.

3. Analysis set

The analysis of the primary and secondary endpoints will include all study participants enrolled in the study. If it is found that the eligibility criteria are not met after the start of the study, they will be excluded from the analysis.

4. Data handling

4.1 Handling of missing data and outlier

Handling of missing data

In case of missing data, no imputation will be performed. However, if it is known before the data are fixed that there are missing values that may have a significant impact on the analysis results, methods to deal with the missing values will be described in this analysis plan.

Handling of outliers

In case of abnormal data, the analysis will be performed without excluding those values. However, if the presence of an outliers that may have a significant impact on the analysis results is known prior to data fixation, methods to deal with the outliers will be described in this analysis plan.

4.2 Allowance range of time-specific measurements

The allowance range listed in the schedule table on page 7 of the study protocol will be adopted.

4.3 Definition of endpoints

- Age is defined as the age at the time of admission.
- The unit of time in the analysis of this study is, in principle, handled in months.

  In this study, one month is approximately 30.4 (=365.25/12) days, and if the acquired data are in dates, the conversion will be performed as follows

  Number of months = number of days * 12 / 365.25
- Time to death, rehospitalization, and unscheduled visits

  Definition of time to death

  The duration of time from the date of enrollment and random allocation to death from any cause.

  Definition of time to rehospitalization

  The duration of time from the date of enrollment and random allocation to "rehospitalization for any new event". A transfer to a hospital is considered a continuation of hospitalization, thus will not be deemed as rehospitalization.

  Definition of time to unscheduled hospital visits

  The duration of time from the date of enrollment and random allocation to "unscheduled hospital visit".

  If two or more of these deaths, rehospitalizations, or unscheduled visits are confirmed, the shortest period of time for each is defined as the "time to death, rehospitalization, or unscheduled visit.

- Body Mass Index (BMI) is calculated based on the following formula

  BMI (kg/m2) = Weight (kg) / Height (m) / Height (m)

- Time to death during initial hospitalization

  Time to death from any cause occurring during the initial hospitalization, starting from the date of enrollment and random allocation.

5. Definition related to statistical analysis

5.1 Definition related to statistical analysis methods

- Descriptive statistics for categorical data will be count and proportion for each category.

- Descriptive statistics for continuous data will be the number of persons, mean, standard deviation, median, 25th percentile, 75th percentile, minimum and maximum.
- Two-sided test with a significance level of 0.05 will be used for group comparison.

5.2 Number of digits displayed

- Proportions (%) are displayed to one decimal place.
- Mean, standard deviation, median, 25th percentile, 75th percentile, minimum, maximum, and confidence interval should be displayed one digit below the number of digits from the input data.

5.3 Software used for analysis

As a rule, use R version 4.0 or higher.

6. Classification of participants

6.1 Classification of participants

A flow diagram for the following items will be provided. The count and proportion of each item will be calculated.

[Classification]

Cases with consent, eligible cases, ineligible cases, enrolled cases, unenrolled cases, cases with completed follow-up, discontinued cases.

6.2 Discontinuation or dropout from the study

Subjects who discontinued or dropped out of the study, along with the reasons for their discontinuation or dropout will be listed.

[Variables]

Subject identification code, date of enrollment, date of discontinuation or dropout, reason for discontinuation or dropout.

7. Participant background

Descriptive statistics for the following variables will be calculated for each group. In addition, a list of the following variables of participant background will be prepared.

[Variables]

Categorical data:

Gender, race, comorbidities, medical history, smoking status, history of falls within 3 months prior to enrollment, level of care required, prescription drug information, current medical history (name of diagnosis), index disease

Continuous data:

Age at admission, height at admission, weight at admission, BMI at admission, vital signs at admission (blood pressure, pulse rate, temperature), laboratory results at admission (serum creatinine level, serum sodium level, serum potassium level, eGFR, pO2, pCO2)

8. Primary endpoint

8.1 Time to rehospitalization, unscheduled visit, or all-cause death

8.1.1 Estimation of Survival Functions

Survival function of the time until rehospitalization, unscheduled visit, or all-cause death will be estimated using the Kaplan-Meier method for each group.

8.1.2 Event rate

Survival rate and its 95% confidence interval at the following time points will be calculated using the survival function estimated in 8.1.1.
[Time point]
6 months
12 months

8.1.3 Between-group comparison

Group comparisons will be made using stratified log-rank tests with stratified age groups (65-74, 75-84, 85+) as the strata.

8.1.4 Hazard ratio

The hazard ratios and 95% confidence intervals for the intervention group compared to the usual care will be calculated using a Cox regression model with the time to rehospitalization, unscheduled visit, or all-cause death as the outcome variable and the allocation group and age group as explanatory variables.

8.1.5 Stratified hazard ratio

The baseline hazard will be stratified based on age group strata, and the hazard ratio and its 95% confidence interval will be calculated for the intervention group compared to the usual care using a Cox regression model with the time until rehospitalization, unscheduled hospital visit, or all-cause death as the outcome variable and the allocation group as the explanatory variable.

8.2 Time to rehospitalization

The same analysis as in 8.1 will be performed for time to rehospitalization.

8.3 Time to unscheduled hospital visit

The same analysis as in 8.1 will be performed for time to unscheduled hospital visit.

8.4 Time to all-cause death

The same analysis as in 8.1 will be performed for time to all-cause death.

8.5 Subgroup analysis according to index diseases

Subgroup analysis based on the index diseases listed below will be performed for the following analyses.

[Index disease]

- Heart failure
- Pneumonia
- Diabetes mellitus
- Cerebral infarction
- Urinary tract infection

8.5.1 Time to first occurrence of any of rehospitalization, unscheduled visit, or all-cause death

8.5.1.1 Estimation of Survival Functions

Survival function of the time until rehospitalization, unscheduled visit, or all-cause death occurs will be estimated by the Kaplan-Meier method for each group.

8.5.1.2 Event rate

The survival rate and its 95% confidence interval at 12 month will be calculated using the survival function estimated in 8.5.1.1.

8.5.2 Time to rehospitalization

The same analysis as in 8.5.1 will be performed for time to rehospitalization.

8.5.3 Time to unscheduled hospital visit

The same analysis as in 8.5.1 will be performed for time to unscheduled hospital visit.

8.5.4 Time to all-cause death

The same analysis as in 8.5.1 will be performed for time to all-cause death.

8.6 Subgroup analysis according to index drug class

The analysis will be conducted in the same method as in 8.5, with subgroups based on the use of the following drug classes at baseline: time to any occurrence of rehospitalization, unscheduled visit, or all-cause mortality; time to rehospitalization; time to unscheduled visit; time to all-cause death.

[Index drug class]
Antiplatelets/anticoagulants
Antihypertensives
Antidiabetics
Sedative

8.7 List of variables related to primary endpoint

The following items related to the primary endpoint will be listed.
[Variables]
Date of enrollment, date of rehospitalization, date of unscheduled visit, date of all-cause death, cause of death, presence of index disease (heart failure, pneumonia, diabetes, stroke, urinary tract infection), presence of index drug class (antiplatelets/anticoagulants, antihypertensives, antidiabetics, sedatives).

9. Secondary endpoints

9.1 Death during initial hospitalization

Survival function for time to death during the initial hospitalization will be estimated using the Kaplan-Meier method for each group. In addition, differences in survival functions between groups will be compared using the Log-rank test.

9.2 Falls and fall-related injuries

The proportion of falls and injuries due to falls at each time point and their 95% confidence intervals will be calculated for each group.

9.3 Unscheduled hospital visit

If a participant visited hospital without appointment, it will be treated as unscheduled visit regardless of the reason for the visit. The rate of unscheduled visit and its 95% confidence interval at each time point will be calculated for each group.

9.4 Health-related quality of life score (EQ-5D Japanese version)

Descriptive statistics for the EQ-5D scores at admission and at 6 and 12 months will be calculated according to group allocation.

9.5 Level of long-term care required

The level of long-term care required at admission, 6 and 12 months after study enrollment, and their 95% confidence intervals will be calculated for each group.

9.6 Number of regularly prescribed medications and drug name

Descriptive statistics of the number of medications taken at admission, discharge, and 6 and 12 months after study enrollment and the proportion of drug names will be calculated for each group.

9.7 Number of potentially inappropriate medications and drug name

The number of potentially inappropriate prescriptions is defined as the number of medications that meet STOPP criteria, and the names of medications that meet STOPP criteria are defined as potentially inappropriate drug names. Descriptive statistics of the number of potentially inappropriate prescriptions and the proportion of potentially inappropriate drug names will be calculated for each group at admission, discharge, and 6 and 12 months after study enrollment.

9.8 Human and time costs of interventions

Descriptive statistics of the human and time costs of the intervention at each time point will be calculated.

10. Safety

10.1 Adverse events

The number of participants in each group who experienced adverse events other than those associated with the primary endpoint and the proportion of such events will be calculated.

10.2 Adverse events according to event type

The number of participants in each group who experienced adverse events other than those associated with the primary endpoint and the proportion of such events will be calculated by adverse event.

10.3 Adverse events according to event type and severity

The number of participants in each group who experienced adverse events other than those associated with the primary endpoint and the proportion of such events by event and severity will be calculated.

10.4 List of adverse events

The list of adverse events other than those associated with the primary endpoint will be created.

10.5 Adverse events associated with the primary endpoint

The number of participants in each group who experienced an event associated with the primary endpoint and the proportion of such events will be calculated.

10.6 Adverse events associated with the primary endpoint according to event type

The number and proportion of participants in each group with each event associated with the primary endpoint will be calculated.

10.7 Adverse events associated with the primary endpoint according to event type and severity

The number and proportion of participants in each group who experienced an event associated with the primary endpoint will be calculated according to type of events and event severity.

10.8 Events associated with primary endpoints

The list of events associated with the primary endpoint will be calculated.

11. Reference

None
