# Supplementary material for: Medication Optimization Protocol Efficacy for Geriatric Inpatients: A Randomized Clinical Trial
Source: JAMA Netw Open. 2024 Jul 30;7(7):e2423544. doi: 10.1001/jamanetworkopen.2024.23544 (PMC11289701; doi:10.1001/jamanetworkopen.2024.23544)
Supplement: Supplement 2. — eMethods eTable 1. Top 10 Regular Medications at Baseline in the Intervention and Usual Care Group eTable 2. Top 10 Potentially Inappropriate Medication at Baseline in the Intervention and Usual Care Group eTable 3. Number of Recommendations and Acceptance Rate According to ATC Classification eTable 4. Per-Protocol Analysis Restricted to Participants in the Intervention Arm Who Were Subjected to One or More Recommendations for Deprescribing and Accepted the Proposal eTable 5. Proportion of Participants With Polypharmacy (5+ Medications), Hyper Polypharmacy (10+ Medications), and Potentially Inappropriate Medication in Both Groups eTable 6. Adverse Events Reported in the Intervention and Usual Care Group eTable 7. Post-Hoc Subgroup Analysis Restricted to Participants in Both Groups With One or More Potentially Inappropriate Medications eFigure 1. Kaplan-Meier Survival Plot of Time to First Fall or Fall-Related Injuries for Participants in the Intervention Group and Usual Care Group: Results From Modified Intention-to-Treat Analysis eFigure 2. Forest Plot of the Primary Outcome According to Subgroups [file jamanetwopen-e2423544-s002.pdf]

Supplemental Online Content

Ie K, Hirose M, Sakai T, et al. Medication optimization protocol efficacy for geriatric inpatients: a randomized clinical trial. *JAMA Netw Open.* 2024;7(7):e2423544. doi:10.1001/jamanetworkopen.2024.23544

eMethods

eTable 1. Top 10 Regular Medications at Baseline in the Intervention and Usual Care Group

eTable 2. Top 10 Potentially Inappropriate Medication at Baseline in the Intervention and Usual Care Group

eTable 3. Number of Recommendations and Acceptance Rate According to ATC Classification

eTable 4. Per-Protocol Analysis Restricted to Participants in the Intervention Arm Who Were Subjected to One or More Recommendations for Deprescribing and Accepted the Proposal

eTable 5. Proportion of Participants With Polypharmacy (5+ Medications), Hyper Polypharmacy (10+ Medications), and Potentially Inappropriate Medication in Both Groups

eTable 6. Adverse Events Reported in the Intervention and Usual Care Group

eTable 7. Post-Hoc Subgroup Analysis Restricted to Participants in Both Groups With One or More Potentially Inappropriate Medications

eFigure 1. Kaplan-Meier Survival Plot of Time to First Fall or Fall-Related Injuries for Participants in the Intervention Group and Usual Care Group: Results From Modified Intention-to-Treat Analysis

eFigure 2. Forest Plot of the Primary Outcome According to Subgroups

This supplemental material has been provided by the authors to give readers additional information about their work.

© 2024 Ie K et al. *JAMA Network Open.*

eMethods

Inclusion criteria

The inclusion criteria for participants:

1. Medical inpatients admitted to the internal medicine departments (general internal medicine, gastroenterology&hepatology, cardiology, pulmonology, metabolism/endocrinology, nephrology, and neurology) at Kawasaki Municipal Tama Hospital;
2. Aged 65 years or older;
3. Taking five or more regularly prescribed medications*;
4. Predicted length of hospital stay after admission: 1 week or longer;
5. Patients who are deemed eligible to take the drug orally by their attending physician;
6. Patients who have been fully informed of the study and who have given their or their surrogate's free and voluntary written consent based on a thorough understanding of the study.

*A regularly prescribed medication is defined as "any form of prescribed oral medications recorded in the participant's medical record handbook, a referral letter, or electronic medical record over 28 days or longer at the time of hospital admission." Drugs that are used "as needed" were not counted as regular medications.

Exclusion criteria

Participants who meet any of the following criteria are not eligible for this study:

1. Attending physicians disagreeing to study participation;
2. Life expectancy of less than 1 month based on their attending physician's clinical judgement.

Primary outcome

The primary outcome is a composite of all-cause death, unscheduled hospital visits, and rehospitalization until 48 weeks after study enrolment. Time to the first occurrence of primary composite outcome was recorded for the survival analysis.

1. **All-cause death**

2. **Unscheduled hospital visits**
   An unscheduled hospital visit is defined as an unexpected visit to the emergency department or outpatient clinic during the follow-up period due to new or worsening symptoms, signs, and concerns.

3. **Rehospitalization**
   Any rehospitalization due to new or worsening symptoms, signs, and concerns after initial hospital discharge. A hospital transfer is deemed as continuation of hospitalization rather than rehospitalization.

Secondary outcomes

Secondary outcomes included time to the first occurrence of each of the primary outcome, all-cause death during initial hospitalization, and falls and fall-related injuries.

1. **All-cause death**

2. **Unscheduled hospital visits**

3. **Rehospitalization**

4. **All-cause death during initial hospitalization**

© 2024 Ie K et al. *JAMA Network Open.*

5. **Fall**
   A fall is defined as 'unintentionally coming to the ground or some lower level and other than as a consequence of sustaining a violent blow, loss of consciousness, sudden onset of paralysis as in stroke or an epileptic seizure' according to Gibson *et al.*'s definition.[1]

In addition, the following outcomes at baseline, 24 weeks, and 48 weeks after study enrolment were recorded.

1. **Number of regularly prescribed medications**
   The number of prescribed medications listed in participant's medical record handbook, referral letter, or electronic medical record with a duration of 28 days or longer at the baseline, 24 weeks, and 48 weeks after study enrolment.

2. **Number of potentially inappropriate medications**
   The number of any regularly prescribed medications listed in the STOPP criteria[2] at the baseline, 24 weeks, and 48 weeks after study enrolment.

3. **Level of long-term care required**
   The level of long-term care required, under the Japanese long-term care insurance system, at the baseline, 24 weeks, and 48 weeks after study enrolment. The levels are assigned by the local government as follows: independent, support required 1 or 2, and care required 1 to 5—where care level 5 implies the highest level of requirement for long-term care and independent implies the lowest level of requirement.[3]

4. **Health-related quality of life**
   Self-reported general health status using EQ5D-3L.[4] We used the Japanese version of EQ5D-3L and a Japanese scoring system that have been found to be valid and reliable.[5]

Adverse events definitions

<u>Adverse events</u>
Any adverse events according to the Japanese version of CTCAE 4.0.[6] Drug names, symptom onset timing, severity, treatment, consequence, and relevance to the intervention are entered in the report form.

<u>Serious adverse events</u>
Serious adverse events in the MPEG trial are defined as follows:
1) All-cause death;
2) All-cause rehospitalization;
3) Disability.

References

1. Gibson MJ, Andres RO, Isaacs B, *et al.* The prevention of falls in later life. A report of the Kellogg International Work Group on the Prevention of Falls by the Elderly. Dan Med Bull. 1987 Apr;34 Suppl 4:1-24.
2. O'Mahony D, O'Sullivan D, Byrne S, O'Connor MN, Ryan C, Gallagher P. STOPP/START criteria for potentially inappropriate prescribing in older people: version 2. Age Ageing. 2015;44(2):213-218.
3. The Japanese Ministry of Health Labour and Welfare. Long-Term care insurance in Japan, 2002. Available: https://www. mhlw. go. jp/ english/topics/ elderly/ care/ index. html [Accessed 22 Jan 2024].
4. EuroQol Group. EuroQol--a new facility for the measurement of health-related quality of life. Health Policy. 1990;16(3):199-208.
5. Nishimura S, Tsuchiya A, Hisashige A, et al. The development of the Japanese EuroQol instrument. Iryo To Shakai 1998;8:109–23.
6. Miyaji T, Iioka Y, Kuroda Y, et al. Japanese translation and linguistic validation of the US National cancer Institute's patient-reported outcomes version of the common terminology criteria for adverse events (PRO-CTCAE). J Patient Rep Outcomes 2017;1:8.

© 2024 Ie K et al. *JAMA Network Open.*

eTable 1: Top 10 regular medications at baseline in the intervention and usual care group.

Drug name	Intervention group (N = 215)	Usual care group (N = 227)	Total (N = 442)
Amlodipine Besilate	84 (39.1%)	76 (33.5%)	160 (36.2%)
Magnesium oxide	62 (28.8%)	53 (23.3%)	115 (26.0%)
Furosemide	48 (22.3%)	52 (22.9%)	100 (22.6%)
Lansoprazole	44 (20.5%)	55 (24.2%)	99 (22.4%)
Aspirin	54 (25.1%)	41 (18.1%)	95 (21.5%)
Esomeprazole magnesium hydrate	39 (18.1%)	33 (14.5%)	72 (16.3%)
Sennoside	37 (17.2%)	32 (14.1%)	69 (15.6%)
Bisoprolol fumarate	27 (12.6%)	37 (16.3%)	64 (14.5%)
Nifedipine	26 (12.1%)	34 (15.0%)	60 (13.6%)
Atorvastatin calcium hydrate	33 (15.3%)	23 (10.1%)	56 (12.7%)

© 2024 Ie K et al. *JAMA Network Open.*

eTable 2: Top 10 potentially inappropriate medications at baseline in the intervention and usual care group[a]

Drug name	Intervention group (N = 215)	Usual care group (N = 227)	Total (N = 442)
Brotizolam	14 (6.5%)	15 (6.6%)	29 (6.6%)
Etizolam	14 (6.5%)	13 (5.7%)	27 (6.1%)
Glimepiride	16 (7.4%)	9 (4.0%)	25 (5.7%)
Zolpidem tartrate	8 (3.7%)	16 (7.0%)	24 (5.4%)
Quetiapine fumarate	2 (0.9%)	10 (4.4%)	12 (2.7%)
Eszopiclone	4 (1.9%)	5 (2.2%)	9 (2.0%)
Alprazolam	7 (3.3%)	2 (0.9%)	9 (2.0%)
Triazolam	7 (3.3%)	2 (0.9%)	9 (2.0%)
Celecoxib	5 (2.3%)	2 (0.9%)	7 (1.6%)
Zopiclone	1 (0.5%)	5 (2.2%)	6 (1.4%)
Tramadol hydrochloride	4 (1.9%)	2 (0.9%)	6 (1.4%)
Risperidone	4 (1.9%)	2 (0.9%)	6 (1.4%)

[a] Potentially inappropriate medication was determined based on STOPP/START criteria version 2[22].

© 2024 Ie K et al. *JAMA Network Open.*

eTable 3: Number of recommendations and acceptance rate according to ATC classification

ATC	Drug class	Recommendation	Acceptance	Acceptance rate (%)
A02BX	Other drugs for peptic ulcer and gastro-oesophageal reflux disease (GORD)	37	33	89.2
A02BC	Proton pump inhibitors	36	12	33.3
C08CA	Dihydropyridine derivatives	27	17	63.0
C10AA	HMG CoA reductase inhibitors	26	20	76.9
N05BA	Benzodiazepine derivatives	26	15	57.7
C09CA	Angiotensin II receptor blockers (ARBs), plain	24	18	75.0
M04AA	Preparations inhibiting uric acid production	24	16	66.7
A06AD	Osmotically acting laxatives	23	10	43.5
B03BA	Vitamin B12 (cyanocobalamin and analogues)	23	16	69.6
-	Kampo formulation for prescription	23	16	69.6
A07FA	Antidiarrheal microorganisms	21	12	57.1
N05CD	Benzodiazepine derivatives	19	11	57.9
A06AB	Contact laxatives	18	11	61.1
A11CC	Vitamin d and analogues	17	9	52.9
A02BA	H2-receptor antagonists	16	9	56.3
B01AC	Platelet aggregation inhibitors excl. heparin.	14	9	64.3
N02BE	Anilide	14	10	71.4
A10BB	Sulfonylureas	13	11	84.6
G04BD	Drugs for urinary frequency and incontinence	13	11	84.6
N05CF	Benzodiazepine related drugs	13	7	53.8
N06DA	Anticholinesterases	12	10	83.3
C03CA	Sulfonamides, plain	11	10	90.9
N02BF	Gabapentinoids	11	9	81.8
A10BH	Dipeptidyl peptidase 4 (DPP-4) inhibitors	10	4	40.0
B03AA	Iron bivalent, oral preparations	10	7	70.0
L01XX	Other antineoplastic agents	10	10	100.0
M01AB	Acetic acid derivatives and related substances	10	8	80.0

ATC=Anatomical Therapeutic Chemical; HMG-CoA=hydroxymethylglutaryl-coenzyme A. Potentially inappropriate medications with 10 or more recommendations from the deprescribing team were listed in the table.

© 2024 Ie K et al. *JAMA Network Open.*

eTable 4: Per-protocol analysis restricted to participants in the intervention arm who were subjected to one or more recommendations for deprescribing and accepted the proposal.

Outcomes	Intervention group, No. (%) (N = 153)	Usual care group, No. (%) (N = 218)	SHR (95% CI)[a]
All-cause death, unscheduled hospital visits, or rehospitalization	76 (49.7)	108 (49.5)	1.05 (0.78-1.41)
All-cause death	34 (22.2)	45 (20.6)	1.13 (0.72-1.76)
Unscheduled hospital visits	55 (35.9)	82 (37.6)	1.00 (0.71-1.41)
Rehospitalization	44 (28.8)	62 (28.4)	1.03 (0.70-1.51)

Abbreviation: SHR, stratified hazard ratio.
[a] Age-group stratified HR (65–74, 75–84 and 85+) was estimated using Cox proportional hazards model.

© 2024 Ie K et al. *JAMA Network Open.*

eTable 5: Proportion of participants with polypharmacy (5+ medications), hyper polypharmacy (10+ medications), and potentially inappropriate medication in both groups

	Intervention group		Usual care group		
	No[a]	No. (%)	No[a]	No. (%)	AOR (95% CI); p value
**Patients with 5+ medications**					
Baseline	215	215 (100)	227	227 (100)	-
Discharge	206	155 (75.2)	218	187 (85.8)	0.44 (0.26-0.74)[b]; p< .01
6 months	177	144 (81.4)	192	171 (89.1)	0.48 (0.26-0.89)[b]; p= .02
12 months	165	140 (84.8)	171	148 (86.5)	0.83 (0.44-1.57)[b]; p= .57
**Patients with 10+ medications**					
Baseline	215	75 (34.9)	227	66 (29.1)	-
Discharge	206	34 (16.5)	218	54 (24.8)	0.36 (0.19-0.67)[b]; p< .01
6 months	177	36 (20.3)	192	55 (28.6)	0.43 (0.24-0.77)[b]; p< .01
12 months	165	40 (24.2)	171	51 (29.8)	0.61 (0.34-1.09)[b]; p= .10
**Patients with 1+ PIM[c]**					
Baseline	215	90 (41.9)	227	97 (42.7)	-
Discharge	206	54 (26.2)	218	72 (33.0)	0.56 (0.33-0.94)[d]; p= .03
6 months	177	49 (27.7)	192	72 (37.5)	0.50 (0.29-0.86)[d]; p= .01
12 months	165	44 (26.7)	171	64 (37.4)	0.45 (0.25-0.80)[d]; p< .01

Abbreviations: AOR, adjusted odds ratio; PIM, potentially inappropriate medications.
[a] Number of participants varied as each was based on available data. [b] Multivariable logistic regression model adjusted for age-group strata and the number of medications at baseline. [c] PIM was determined based on STOPP/START criteria version 2[22]. [d] Multivariable logistic regression model adjusted for age-group strata and the number of PIMs at baseline.

© 2024 Ie K et al. *JAMA Network Open.*

eTable 6: Adverse events reported in the intervention and usual care group.

Adverse events	Intervention group (n=215) No. (%)	Usual care group (n=227) No. (%)
Falls	33 (15.3)	38 (16.7)
Lung infection	17 (7.9)	16 (7.0)
Heart failure	10 (4.7)	14 (6.2)
Fever	10 (4.7)	11 (4.8)
Bone fracture	5 (2.3)	8 (3.5)
Death due to senility	5 (2.3)	7 (3.1)
Stroke	6 (2.8)	4 (1.8)
Dyspnea	4 (1.9)	4 (1.8)
Chest pain	5 (2.3)	2 (0.9)
Urinary tract infection	2 (0.9)	5 (2.2)
Liver cancer	3 (1.4)	4 (1.8)
Pancreatic cancer	4 (1.9)	3 (1.3)
Aspiration	2 (0.9)	4 (1.8)
Syncope	3 (1.4)	2 (0.9)
Sepsis	2 (0.9)	3 (1.3)
Respiratory failure	0 (0.0)	4 (1.8)
Swelling of limbs	2 (0.9)	2 (0.9)
Herpes Zoster	2 (0.9)	2 (0.9)
Altered mentation	2 (0.9)	2 (0.9)
Chronic kidney disease	1 (0.5)	3 (1.3)
Others	78 (36.3)	87 (38.3)

© 2024 Ie K et al. *JAMA Network Open.*

eTable 7: Post-hoc subgroup analysis restricted to participants in both groups with one or more potentially inappropriate medications[a]

Outcomes	Intervention group, No. (%) (N = 90)	Usual care group, No. (%) (N = 97)	SHR (95% CI)[b]
All-cause death, unscheduled hospital visits, or rehospitalization	46 (51.1)	54 (55.7)	0.96 (0.65-1.43)
All-cause death	20 (22.2)	19 (19.6)	1.25 (0.67-2.35)
Unscheduled hospital visits	34 (37.8)	40 (41.2)	0.99 (0.63-1.57)
Rehospitalization	26 (28.9)	28 (28.9)	1.09 (0.64-1.87)

Abbreviation: SHR, stratified hazard ratio.
[a] Potentially inappropriate medication was determined based on STOPP/START criteria version 2[22].
[b] Age-group stratified HR (65–74, 75–84 and 85+) was estimated using Cox proportional hazards model.

© 2024 Ie K et al. *JAMA Network Open.*

eFigure 1: Kaplan-Meier survival plot of time to first fall or fall-related injuries for participants in the intervention group and usual care group: results from modified intention-to-treat analysis.

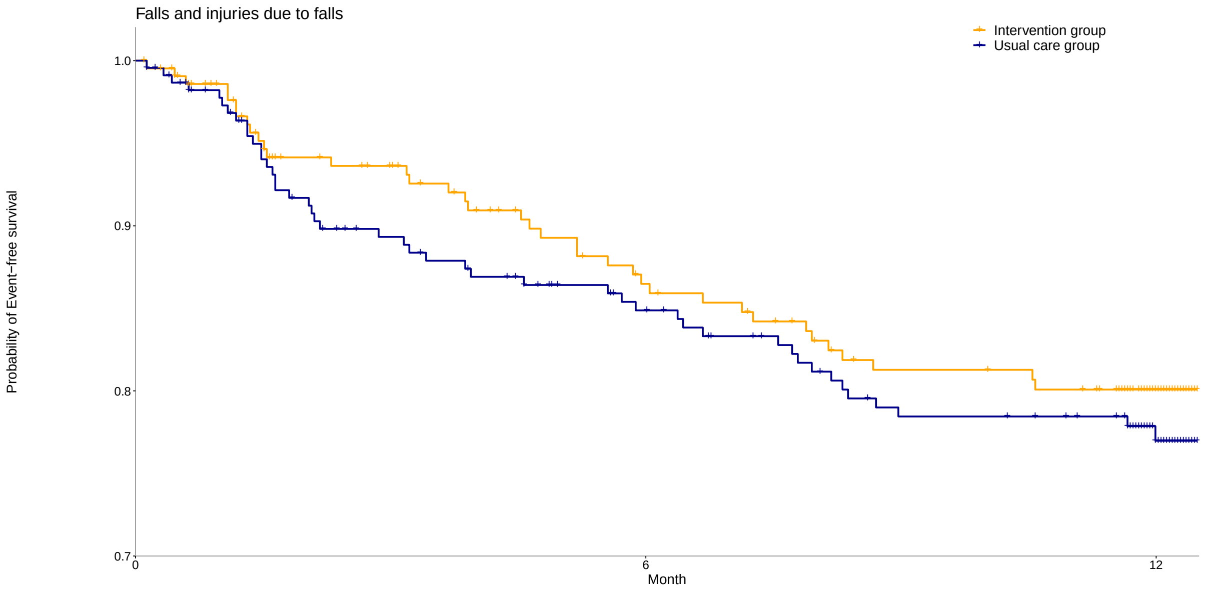

Number at risk

	0	6	12
Intervention group	215	153	84
Usual care group	227	165	84

Tick marks indicate censoring events.

© 2024 Ie K et al. *JAMA Network Open.*

eFigure 2: Forest plot of the primary outcome according to subgroups.

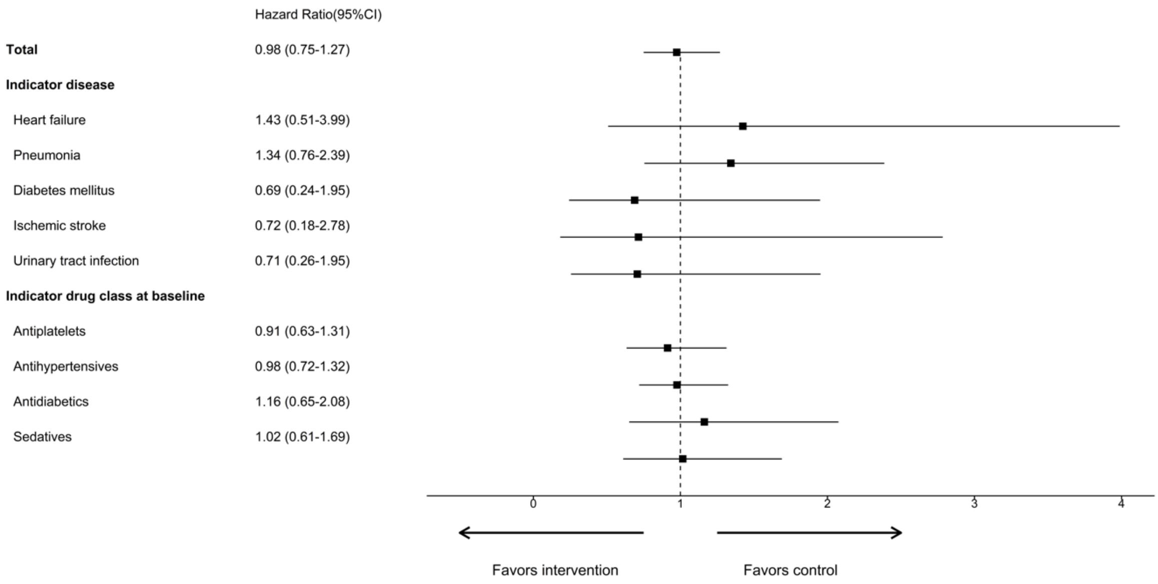

Abbreviation: CI, confidence interval.
Age-group stratified hazard ratio (65–74, 75–84 and 85+) was estimated using Cox proportional hazards model.

© 2024 Ie K et al. *JAMA Network Open.*
