# Supplementary material for: Medication Optimization Protocol Efficacy for Geriatric Inpatients: A Randomized Clinical Trial
Source: JAMA Netw Open. 2024 Jul 30;7(7):e2423544. doi: 10.1001/jamanetworkopen.2024.23544 (PMC11289701; doi:10.1001/jamanetworkopen.2024.23544)
Supplement: Supplement 3. — Data Sharing Statement [file jamanetwopen-e2423544-s003.pdf]

Data Sharing Statement

Ie. Medication Optimization Protocol Efficacy for Geriatric Inpatients. *JAMA Netw Open*. Published July 30, 2024. doi:10.1001/jamanetworkopen.2024.23544

Data
Data available: Yes
Data types: Deidentified participant data
How to access data: The datasets generated during the study will be available from the corresponding author upon reasonable request. kenye.ie@marianna-u.ac.jp
When available: With publication

Supporting Documents
Document types: Statistical/analytic code
How to access documents: The trial protocol, including the statistical analysis plan, will be made available as online supplements.
When available: With publication

Additional Information
Who can access the data: Researchers whose proposed use of the data has been approved.
Types of analyses: For any purpose if the proposed use of the data has been approved.
Mechanisms of data availability: The datasets generated during the study will be available from the corresponding author upon reasonable request, after approval of a proposal.
